# Supplementary material for: Strengthening integration of chronic care in Africa: protocol for the qualitative process evaluation of integrated HIV, diabetes and hypertension care in a cluster randomised controlled trial in Tanzania and Uganda
Source: BMJ Open. 2020 Oct 7;10(10):e039237. doi: 10.1136/bmjopen-2020-039237 (PMC7542920; doi:10.1136/bmjopen-2020-039237)
Supplement: Author's manuscript [file bmjopen-2020-039237.draft_revisions.pdf]

BMJ Open

BMJ Open is committed to open peer review. As part of this commitment we make the peer review history of every article we publish publicly available.

When an article is published we post the peer reviewers' comments and the authors' responses online. We also post the versions of the paper that were used during peer review. These are the versions that the peer review comments apply to.

The versions of the paper that follow are the versions that were submitted during the peer review process. They are not the versions of record or the final published versions. They should not be cited or distributed as the published version of this manuscript.

BMJ Open is an open access journal and the full, final, typeset and author-corrected version of record of the manuscript is available on our site with no access controls, subscription charges or pay-per-view fees (http://bmjopen.bmj.com).

If you have any questions on BMJ Open's open peer review process please email info.bmjopen@bmj.com

BMJ Open

Strengthening integration of chronic care in Africa: protocol for the qualitative process evaluation of the integrating and decentralising HIV, diabetes and hypertension cluster randomised controlled trial in Uganda and Tanzania

Journal:	*BMJ Open*
Manuscript ID	bmjopen-2020-039237
Article Type:	Protocol
Date Submitted by the Author:	09-Apr-2020
Complete List of Authors:	Van Hout, Marie-Claire; Liverpool John Moores University, Public Health Institute<br>Bachmann, Max; University of East Anglia Faculty of Medicine and Health Sciences, Norwich Medical School<br>Lazarus, Jeffrey; Instituto de Salud Global de Barcelona, Hospital Cliínic, University of Barcelona;<br>Shayo, Elizabeth<br>Bukenya, Dominic<br>Picchio, Camila; Barcelona Institute for Global Health (ISGlobal), Hospital Cliníc, University of Barcelona, Barcelona, Spain<br>Nyirenda, Moffat; London School of Hygiene and Tropical Medicine,<br>Mfinanga, Sayoki<br>Birungi, Josephine<br>Okebe, Joseph<br>Jaffar, Shabbar; Liverpool School of Tropical Medicine, Department of International Public Health
Keywords:	Hypertension < CARDIOLOGY, DIABETES & ENDOCRINOLOGY, International health services < HEALTH SERVICES ADMINISTRATION & MANAGEMENT, Organisation of health services < HEALTH SERVICES ADMINISTRATION & MANAGEMENT

SCHOLARONE™
Manuscripts

I, the Submitting Author has the right to grant and does grant on behalf of all authors of the Work (as defined in the below author licence), an exclusive licence and/or a non-exclusive licence for contributions from authors who are: i) UK Crown employees; ii) where BMJ has agreed a CC-BY licence shall apply, and/or iii) in accordance with the terms applicable for US Federal Government officers or employees acting as part of their official duties; on a worldwide, perpetual, irrevocable, royalty-free basis to BMJ Publishing Group Ltd ("BMJ") its licensees and where the relevant Journal is co-owned by BMJ to the co-owners of the Journal, to publish the Work in this journal and any other BMJ products and to exploit all rights, as set out in our licence.

The Submitting Author accepts and understands that any supply made under these terms is made by BMJ to the Submitting Author unless you are acting as an employee on behalf of your employer or a postgraduate student of an affiliated institution which is paying any applicable article publishing charge ("APC") for Open Access articles. Where the Submitting Author wishes to make the Work available on an Open Access basis (and intends to pay the relevant APC), the terms of reuse of such Open Access shall be governed by a Creative Commons licence – details of these licences and which Creative Commons licence will apply to this Work are set out in our licence referred to above.

Other than as permitted in any relevant BMJ Author's Self Archiving Policies, I confirm this Work has not been accepted for publication elsewhere, is not being considered for publication elsewhere and does not duplicate material already published. I confirm all authors consent to publication of this Work and authorise the granting of this licence.

For peer review only

Title

Strengthening integration of chronic care in Africa: protocol for the qualitative process evaluation of the integrating and decentralising HIV, diabetes and hypertension cluster randomised controlled trial in Uganda and Tanzania

Author Listing

Marie Claire Van Hout, Liverpool John Moores University, United Kingdom

Max Bachmann, University of East Anglia, United Kingdom

Jeffrey V Lazarus, Barcelona Institute for Global Health (ISGlobal), Hospital Clínic, University of Barcelona, Spain

Elizabeth H. Shayp, National Institute for Medical Research, Tanzania

Dominic Bukenya, MRC/UVRI/LSHTM Uganda Research Unit, Uganda

Camila A Picchio, Barcelona Institute for Global Health (ISGlobal), Hospital Clínic, University of Barcelona, Spain

Moffat Nyirenda Medical Research Council Uganda, Uganda

Sayoki Mfinanga, National Institute for Medical Research and Liverpool School of Tropical Medicine, Liverpool, United Kingdom, and Muhimbili University of Health and Allied Sciences, Tanzania

Josephine Birungi, Medical Research Council/Uganda Virus Research Institute & London School of Hygiene and Tropical Medicine (MRC/UVRI &LSHTM), Uganda

Joseph. Okebe, Liverpool School of Tropical Medicine, United Kingdom

Shabbar Jaffar, Liverpool School of Tropical Medicine , United Kingdom

Corresponding Author:

Professor Marie Claire Van Hout, Public Health Institute, Exchange Station, Liverpool John Moores University, Liverpool L32ET, United Kingdom. Email: m.c.vanhout@ljmu.ac.uk

Article Type: Protocol

For peer review only - http://bmjopen.bmj.com/site/about/guidelines.xhtml

Abstract

Introduction In sub-Saharan Africa (SSA), the burden of non-communicable diseases (NCDs), particularly diabetes mellitus (DM) and hypertension, has increased rapidly in recent years, although HIV infections remain a leading cause of death among young-middle aged adults. Health service coverage for NCDs remains very low in contrast to HIV, despite increasing prevalence of co-morbidity of NCDs with HIV. There is an urgent need to expand healthcare capacity to provide integrated services to address these chronic conditions.

Methods and Analysis: This protocol describes procedures for a qualitative process evaluation of the INTE-AFRICA, a cluster-randomised trial comparing integrated health service provision for HIV-infection, DM and hypertension, to the current stand-alone vertical care. Interviews, focus group discussions and observations of consultations and other care processes in two clinics (Tanzania, Uganda) will be used to explore the experiences of stakeholders (service users, policy makers, healthcare providers, community leaders and members, researchers, non-governmental (NGO) and international organisations) during implementation alongside an understanding of the impact of broader structural and contextual factors.

Ethics and Dissemination: Ethical approval has been granted by Liverpool School of Tropical Medicine (UK), the National Institute of Medical Research (Tanzania) and Medical Research Council (Uganda). The evaluation will provide the opportunity to document implementation of integration over several time points (six, 12 and 18 months) and refine integrated service provision prior to scale-up. This synergistic approach to evaluate, understand and respond will support service integration and inform monitoring, policy and practice development efforts to involve and educate communities in Uganda and Tanzania. It will create a model of care and a platform of best practices and lessons learnt for other countries implementing integrated and decentralised community health services.

Key Words

Health service delivery, Non communicable disease, HIV, integration, task-shifting, sub-Saharan Africa

Article Summary: Strengths and Limitations of this study

- Whilst HIV continues to be the leading cause of death among young-middle aged adults in sub-Saharan Africa (SSA), the burden of non-communicable diseases (NCDs) has increased rapidly in recent years, particularly diabetes mellitus and hypertension,
- There is an urgent need to expand the capacity of healthcare systems to manage NCDs, either alongside or integrated with HIV.
- Implementation of INTE-AFRICA in Tanzania and Uganda is complex, requiring concurrent process evaluation to understand and document stakeholders experiences, attitudes, and practices during implementation, and to understand the impact of structural and contextual factors on implementation.
- Limitations of the process evaluation may centre on patient drop out, characteristics of the two selected sites and other external mitigating factors.

For peer review only - http://bmjopen.bmj.com/site/about/guidelines.xhtml

BACKGROUND

The non-communicable disease (NCD) burden is increasing globally and poses a serious public health threat, with type 2 diabetes mellitus (DM) and hypertension contributing to about 9.4 million deaths annually,[1-3] in addition to long-term illness and disability. Over three-quarters of global NCD mortality and the majority of premature deaths occur in low- and middle-income countries (LMICs).[4 5] The International Diabetes Federation has projected that incidence of DM will increase from 415 million in 2015 to 642 million by 2040, and over 70% of cases will be in LMICs.[6] People living with HIV may have increased risks of developing hypertension and DM, with prevalence rates exceeding 5% and 20-30%, respectively.[7] HIV/AIDS remains a major cause of morbidity and mortality among young-middle aged adults in sub-Saharan Africa (SSA), but the region is also experiencing a rapidly rising burden of NCDs (particularly DM and hypertension), rise to a dual HIV-NCD epidemic.[8-10] While lifestyle changes associated with urbanisation and globalization (such as eating habits and lack of physical exercise) underpin the ongoing demographic and epidemiologic transition toward increasing NCDs, these changes also effect chronic infections (such as HIV), resultant inflammation and antiretroviral therapy [8-10] About 65% of people estimated to be living with HIV-infection in Africa are in regular HIV care; and this number is rising.[11] Chronic conditions requiring lifelong regular care, however, have an equal or higher burden than acute infections, with hypertension representing the single largest risk factor for death, and DM which has seen a massive increase in prevalence in a short period of time.[1 9 12 13 1] Patient populations are increasingly demonstrating younger age of onset, and comorbidity conditions of NCDs with HIV, and the impact of NCDs is particularly severe in populations affected by poverty in SSA.[10 15-18]

The majority of the healthcare systems in LMICs, particularly those in Africa, are already overburdened. Moreover, they are ill-prepared to provide chronic care required for most NCDs, since they were designed for episodic care to manage acute infection (e.g. malaria). However, since 2003, significant global investment and development partner engagement has facilitated the establishment of HIV screening and treatment programmes as the first large-scale chronic disease initiatives in Africa. Within these programmes, health services for HIV are typically stand-alone and vertically delivered. They are also often combined with decentralisation and task-shifting, which has enabled primary health centres to treat large numbers of patients while allowing non-clinically qualified health workers to play a major role in supporting patients on HIV treatment. In contrast, health service coverage for NCDs remains very low.[9 19] Coordinated national NCD control and care programmes are relatively new with significant gaps in funding and operational evidence for program implementation.[18 20.] For example, fewer than 5% of persons with DM are thought to be in regular diabetes care, and the figure is likely to be similar for persons with hypertension.[9 15 18] For those in care, health service provision for the diagnosis and management of diabetes and hypertension is insufficient.[9 19 21-24] The expansion of and improvements in antiretroviral therapy (ART) has decreased HIV-related morbidity and

For peer review only - http://bmjopen.bmj.com/site/about/guidelines.xhtml

mortality and contributed to increased survival rate. However, this population is now at risk of developing NCDs, which will diminish the gains from the scale-up of HIV care services.[25]

It is important to learn from and leverage HIV services to improve care for persons with NCDs. There is an urgent need to expand the capacity of healthcare systems to provide services for NCDs, either alongside, or preferably integrated, with HIV.[26] DM, hypertension and HIV-infection require lifelong care. Key challenges in Africa are linkage and retention in care, access and adherence to ART and promotion of healthy behaviours.[27 28] Addressing these challenges requires a common approach, yet current services are primarily stand alone and vertically delivered in clinics within district hospitals or larger primary care facilities. Additionally, duplication of services and crippling costs of accessing health services for patients (typically 10-30% of monthly income) are major factors in patient drop-out. As such, the infrastructure and lessons learnt from the HIV chronic disease prevention, testing and care model can serve as important resources for the expansion of NCD prevention, care and treatment. HIV chronic care management pathways, resources and infrastructure can be leveraged to integrate with newly developing NCD services, which would strengthen the platform for NCD services delivery.[29-31] Health systems have developed experience managing HIV as a chronic disease, including linking and retaining patients in care and supporting treatment adherence in drugs, diagnostics procurement and other key health systems indicators.[28 32-34] Integration of services would therefore reduce duplication and fragmentation of services and could mean that these practices can be applied quickly to NCD control. This will enable effective management of DM and hypertension, prevention of complications, cost savings to the health system and greater access to services and clinical management (e.g. drug-drug interactions, adherence and support) for co-morbid or multi-morbid patients. See **Figure One**. HIV, diabetes and hypertension are common and many people in communities will have two or more chronic conditions, known as multi-morbidity. For these individuals, a single integrated clinic that can cater for multiple (common) conditions would be hugely beneficial both in terms of them accessing health care and also in considering clinical management (e.g. drug-drug interactions, adherence and support).

Insert **Figure One 'Potential benefits of integrating diabetes, hypertension and HIV services for a) DM and hypertension control, and b HIV control'** about here

Despite the increase in academic and clinical interest in HIV/NCD integration in SSA, [16 33 35-40] there remains little evidence about integration in terms of both scope and generalizability as evidence is limited to small scale feasibility studies in largely different contexts. [41-47] The lack of large scale or randomized and/or controlled evaluations and context-specific clinical, cost effectiveness and process outcomes data constrains policymaking and development of integrated care models, which strengthen health systems and are tailored to the distinct needs of each specific SSA country. SSA policy

For peer review only - http://bmjopen.bmj.com/site/about/guidelines.xhtml

frameworks identify three forms of integration: i) integration of HIV/AIDS and NCD responses into the health system, ii) integration within HIV/AIDS or NCD response (intra-), and iii) integration between HIV/AIDS and NCD responses (inter-), and within the latter, *full* integration of HIV/NCD care services into a chronic disease clinic; *partial* integration of certain aspects of NCD care such as screening for NCD risk factors into HIV interventions; or *adaptation* of HIV strategies, systems and tools for NCD care without actual service integration.[26 28 44] There is however a lack of large scale or randomized and/or controlled evaluations, as well as a lack of context specific clinical, cost effectiveness and process outcomes data. This evidence is paramount to inform policy, government resource prioritisation, and to develop integrated care models which strengthen health systems best suited to the needs of that specific SSA country. We present here on the qualitative process evaluation protocol designed to evaluate INTE-AFRICA, a European Commission Horizon 2020 funded implementation research project (protocol number 19-100) operating in Uganda and Tanzania.

The INTE-AFRICA Project

The aim of INTE-AFRICA is to implement and assess the effectiveness of integration of HIV, DM and hypertension services at the point of service delivery covering many health facilities where common approaches to clinical decision-making, drug procurement and human resource management occur. The project will generate the research evidence needed by the health service in Uganda, Tanzania and potentially the rest of Africa, to scale-up and sustain the chronic disease management and services in an integrated manner. We recognise that stigma and discrimination associated with HIV may prevent people from accessing health services.[48] Likewise, persons with DM and hypertension may be reluctant to attend clinics where HIV-infected persons are seeking care either because of the stigma associated with HIV-infection or for other reasons – e.g. because of fear of contracting infection. Nonetheless, managing HIV-infection, like any other chronic condition, could reduce the stigma, thus facilitating increased access to HIV services as well as improved NCD control.

Tanzania and Uganda were specifically chosen as they are low-income and their public and private health facilities are strongly committed to providing services for NCD. However, their health systems struggle to scale-up provision for diabetes and hypertension in the face of competing health demands, including HIV-infection. See **Table One**. Tanzania and Uganda also share relevant characteristics with other low-income countries, especially in sub-Saharan Africa. The process evaluation has the potential to enhance the generalisability of integrated care to other similar settings by providing understanding of the determinants and mechanisms of the implementation process.

Insert **Table One 'Key data on the country settings'** about here

For peer review only - http://bmjopen.bmj.com/site/about/guidelines.xhtml

Our programme is underpinned by a participatory, multi-actor approach which supports dialogue, knowledge exchange, fosters mutual understanding and provides input in policy agendas around diagnosis and treatment of DM, hypertension and HIV in an integrated clinic. The INTE-AFRICA conceptual framework is illustrated in **Figure Two.**

Insert **Figure Two 'INTEAFRICA Conceptual Model'** about here

INTE-AFRICA will test integrated health services at primary care centres for HIV-infection, diabetes and hypertension, by providing a "*One stop shop*" integrated care clinic for these conditions (the intervention). We will conduct a pragmatic parallel arm cluster-randomised trial: 32 urban health facilities in the two countries will be randomised, with 16 facilities allocated to deliver the intervention immediately (intervention arm), and 16 facilities to continue with usual care (control arm). In both arms, the clinic staff will receive refresher training in DM and hypertension management to enable all healthcare staff to deliver the same standard of care to all patients. At each selected facility, cohorts of approximately 220 patients; 110 HIV infected and 110 NCD patients, will be enrolled to evaluate the primary outcomes. Both integrated and vertical health delivery approaches will run concurrently. The primary care facilities will initiate and stabilise patients on treatment, manage complications (including referral to higher facilities) and conduct clinical and laboratory monitoring of patients for all three conditions. Specific characteristics of integrated care in each clinic are: '*One stop shop'* with concurrent management of HIV, hypertension and DM in the same facility; management of patients with HIV, hypertension and DM by the same clinician or team of clinicians (nurses, counsellors other staff); integrated training of clinicians; single waiting area and queue; integrated health education about all three conditions; one pharmacy with a single drug dispensing point; similar testing, and cross-testing, for diagnosis and monitoring with requisition of laboratory tests in the same place; and similar format of paper medical records for each condition kept in the same patient folder. Patients who decline to participate in the research and trial participants in control arm clinics will continue to receive standard vertical health care delivery. Inclusion criteria for participation are: being over 18 years old; having confirmed HIV-infection, diabetes or hypertension; living within the catchment population of the health facility; likely to remain in the catchment population for six months and willing to provide written informed consent. Very ill patients requiring in-patient care will be excluded. Participants will be observed by the research team during clinic visits with additional reviews at six and 12 months. Further details are provided in the trial protocol.

Qualitative Process Evaluation of the INTE AFRICA trial

Process evaluations typically evaluate how and whether interventions are delivered as intended and whether such implementation is congruent with the theory underpinning the intervention.[32 50-52] Updated Medical Research Council (MRC) guidance for evaluation of complex health interventions

For peer review only - http://bmjopen.bmj.com/site/about/guidelines.xhtml

has recently recognised the value of process evaluation within trials stating, "*it can be used to assess fidelity and quality of implementation, clarify causal mechanisms and identify contextual factors associated with variation in outcomes.*" [32]The process evaluation in INTE-AFRICA is particularly focused on context, description of the intervention and its causal assumptions, implementation, mechanisms of impact and outcomes. [32] Further we evaluate the extent to which resources and activities supporting the intervention function to deliver intended outputs, with subsequent improvements in outcomes. A central focus lies in identifying contextually relevant strategies for successful implementation, and practical difficulties in adoption, delivery and maintenance to inform wider implementation.[53] We recognise that outcomes (e.g. knowledge gained) of INTE-AFRICA are dependent on understanding cultures and contexts of the stakeholders (i.e. service users, healthcare providers, policy makers, community leaders/members, non-governmental organisations (NGO), international organisations, and clinical researchers) involved and those surrounding service design and delivery as well as care seeking practices. The role of social and behavioural science approaches is central to this process evaluation. It aims to enhance understanding of issues related to the NCD and HIV agendas and service delivery approaches from all perspectives and stakeholders along the integration process. INTE-AFRICA will utilise a broad social behavioural approach to support community engagement in research in Uganda and Tanzania, going beyond clinical trial recruitment and retention to improve NCD/ HIV literacy. Behavioural and social science research strategies [54] will allow NCD and HIV related research to place the needs and perspectives of people living with NCD and/or HIV at the centre of the progression of clinical studies, such as INTE-AFRICA, and investigate participant motivations and decision-making processes related to preferences for or participation in different types of integrated and decentralised services.

Process evaluation design, like healthcare interventions, requires a theoretical framework to structure the evaluation across sites. We draw here on Bronfenbrenner's ecological model of behaviour [55 56] to conceptualise integrated care as events that disrupt complex social systems[57] operating across multiple contextual levels. We are interested in better understanding the importance of context, which becomes especially relevant when comparing Tanzania and Uganda. According to the Bronfenbrenner model, contextual factors are evaluated at the ***macro*** (universal vs partial coverage; free primary healthcare and drugs, and users fees; fiscal policy); ***meso*** (drug ordering and drug delivery systems, health worker education and employment, community understanding and perspectives on multi-morbidity); and ***micro*** (clinic level management, resources, service user experience) levels. See **Figure Three.** These contextual factors, likely to influence implementation and their effects, will be investigated to capture variation in adoption, delivery and maintenance outcomes as well as responses to the intervention. This will affect both reach and fidelity, which are hypothesised to be important factors in outcome differences.

For peer review only - http://bmjopen.bmj.com/site/about/guidelines.xhtml

Insert **Figure Three 'Potential contextual influences on INTE AFRICA programme implementation cascade'** about here

To situate INTE-AFRICA within this theoretical framework, we will develop a logic model. This model will set out contextual determinants of HIV and NCD illness management in SSA and assess how integrated care components function to address these determinants to improve outcomes in the management of patients. See **Table Two.**

Insert **Table Two 'Logic Model of programme inputs, processes and outcomes'** about here

METHODS

Overall Design

This protocol describes procedures for a qualitative process evaluation of the INTE-AFRICA pragmatic parallel arm cluster-randomised trial comparing integrated health service provision for HIV-infection, DM and hypertension with the current standard vertical care delivery model. The aims of this qualitative process evaluation are to explore the experiences, attitudes and practices of a wide variety of stakeholders during the process of programme implementation and to develop an understanding of the impact of broader structural and contextual factors on the implementation process. The process evaluation works in tandem with the collection of selected clinical outcomes (e.g. clinical efficacy of different treatments) and health economic data (e.g. costs and benefits of different approaches) to estimate the potential benefits to patients and health services at clinic and country level (protocol reported elsewhere). It will use three qualitative research techniques: in-depth interviews with stakeholders (service user, healthcare provider, policy maker, non-governmental organisation (NGO)/international organisation, and clinical researcher); focus group discussions (FGD) with community leaders and community members; and clinic-based observations in one site per country. This design permits an assessment of the fidelity of INTE-AFRICA's implementation, a detailed description of the processes, relationships, and contexts involved in the delivery of integrated care, and the identification of factors attributing to the failure or success of the programme. It thus addresses the *'black box'* problem in interpreting trial results by improving understanding of the mechanisms that connect particular intervention components to particular outcomes.[58] The chosen approach will enhance social construction and acceptability of chosen decentralised integrated approaches, link outcomes to policy and advocacy and impact sustainability of HIV, DM and hypertension chronic disease service integration in two LMIC countries. It provides the opportunity to document and refine INTE-AFRICA activities prior to a larger pragmatic trial or scale-up by Uganda and Tanzanian governments. These synergistic approaches to evaluate, understand and respond will support integration, inform surveillance, policy and practice development and improve efforts to involve and educate communities in Uganda and Tanzania. It will create a model and a platform of

For peer review only - http://bmjopen.bmj.com/site/about/guidelines.xhtml

For peer review only

best practices and lessons learnt for other countries implementing integrated and decentralised community health services for HIV and chronic disease. The process evaluation methods for each objective are described in **Table Three**, including how each method maps onto the three different contextual levels.

Insert **Table Three: 'Process Evaluation Design and data collection framework'** about here.

Public and Patient Involvement

Public and patient involvement (PPI) throughout a programme of research enhances research quality and relevance by providing different perspectives and a sense of ownership. This protocol will adhere to the same principles, and will allow the voice of 'service users' and those affected to be heard, and utilised. Key stakeholders such as service users and their families will be fully involved in guiding the research, acting as research participants, and in implementation of change in health service delivery and integrated care planning. All aspects of the process evaluation are underpinned by participatory action health research and its success and usefulness will be grounded in PPI, participation and engagement in the form of patient/professional identification of research priorities, collaborations and partnerships, expert steering, community participation around health needs and optimal integrated services, awareness raising activities, development of print materials, toolkits and training for healthcare professionals.

Data collection

We will use the Empirical Phenomenological Psychological (EPP) five-step method,[59] which combines psychological, interpretative and idiographic components, to gain understanding of the complex social processes, of social, aged, gendered and culturally/community specific meanings and of incremental understanding of the distinct lived experience of policy makers, service users, health care providers, researchers and communities. We will balance the description of phenomena with interpretation of insights and are cognisant of participant experiential phenomena and authors' interpretation of associated meanings. It will yield an in-depth socio-cultural understanding of service user/participant reported outcomes, their motivations, preferences, beliefs, expectations, identities, hopes and views on conditions, related stigma and of decision-making processes. This will provide better understanding of stakeholder and community positioning during integration. This understanding will inform policy and practice, ensure effective service user education, position service users and their families to understand these conditions and interpret study outcomes and facilitate future HIV and chronic disease clinical studies.

Both descriptive patient level data and rich socio-behavioural qualitative data will be collected by a team of trained researchers in Tanzania and Uganda. Data collection will entail exploring the

For peer review only - http://bmjopen.bmj.com/site/about/guidelines.xhtml

experiences, attitudes and practices of a wide variety of stakeholders during the process of INTE-AFRICA programme implementation and will develop an understanding of the impact of broader structural and contextual factors on the implementation process. [17 53]We will collect data on social behavioural and cultural aspects impacting implementation (e.g. individual and community health risks, protective behaviours and health responses) within the broader social and political frameworks, the practicalities of accessing, providing and sustaining integrated services (e.g. staff time, resources, equity of access, catchment area/populations, quality of care, waiting room dynamics, record keeping and retention across multi/co-morbidities, training gaps); and process indicators (e.g. perceived stigma, acceptability of vertical versus integrated service designs, lay knowledge and awareness and bottlenecks to accessing services). We will also describe implementation of the intervention in terms of fidelity to the intended model of care, adaptations to the intervention during implementation, and dose and reach of intervention components actually delivered and received (such as numbers and proportions of eligible staff who received integrated care training, numbers of proportions of patient participants who received all or most of their care from integrated services, and frequency of drug stock-outs) [69]. The latter data will be complemented by routinely collected quantitative data such as training attendance and medical records. We will document changes in healthcare provider roles, attitudes and patient relationships.

Interviews with service users and providers will include specific questions about their experience and management of individuals with multi-morbid HIV, hypertension and/or diabetes. These include service user, healthcare provider, and policymaker/senior manager perceptions of INTE-AFRICA; impacts of INTE-AFRICA on the provision of integrated HIV/AIDS care and NCD care, and relationships with NGO and international organisations; changes in health provider roles, attitudes, and patient relationships; impacts of the INTE-AFRICA implementation context on trial and health economic cost outcomes; impacts of the INTE-AFRICA intervention on an integrated health systems approach to care (medicine supplies, record keeping, service user education, clinical care pathways, data management, staff training); and barriers to and facilitators of change. The purposive samples of service users and providers (**Table 3**) will include those who live with or manage multi-morbidity. Selected patient outcomes will also be collected in order to estimate the potential benefits to patients and to health services. We will assume a more pragmatic approach when garnering perspectives from higher level stakeholders involved in health policy and practice generation, and NGO and international organisations (e.g. WHO Country offices, UNAIDS, PEPFAR, CDC) providing peripheral supports and guidance.

The data, when combined and triangulated across these stakeholder perspectives, will provide a *'thick description*', of how the intervention was delivered, maintained and experienced by stakeholders.[17 53] It will also offer explanations for observed variation over time and between countries, and detailed

For peer review only - http://bmjopen.bmj.com/site/about/guidelines.xhtml

insight into the interaction between different contextual features and components of integration of NCD or HIV/NCD services. It will also facilitate triangulation of information across stakeholders, clinics and countries. We envisage returning to these two clinics in the future, four years and six years after integration, to achieve a deeper understanding of processes and patient and provider experiences.

Setting, study populations, and recruitment

This will be a cohort study taking place in one site per country, with the same participants interviewed at each time. 25 patients in each facility and 20 healthcare workers (10 in each facility) will be purposely selected and invited; at six, 12 and 18 months after the start of the trial, to reflect retrospectively on their experience of integration. See **Table Three**. These numbers are expected to reach saturation (i.e. the point that further information does not provide any additional variation in observed themes). We may replace participants if there is significant loss to follow up or refusal for repeated interviews. For instance, if a participant drops out at 12 months, we still have their six-month experience documented, and we can replace with a new participant, invited to reflect on their 12-month retrospective experience. Where possible we will gender match interviewers with participants (particularly patients). The following recruitment procedures will take place at each clinic.

- Observations will be made in the *clinic* in consultation with the health facility and clinics in-charges.
- Recruitment of *service users* will be supported by clinic nurses who will identify and approach selected participants who have a minimum of six months experience of integration, and will be asked to consent to partake in an in-depth interview on the day they attend the clinic. INTE-AFRICA researchers will purposively sample women and men of different ages and explore any age/gender and condition ((HIV/hypertensive/diabetic/multi/co-morbid) related differences.
- *Health care providers* at the clinic (health facility managers and health professionals such as physicians, nurses, public health nurses) will be approached at the clinic to participate in an in-depth interview on the day the INTE-AFRICA team are scheduled to attend the clinic.
- *Ministerial policy makers and provincial/regional/district level clinical/health senior management* (directors for NCD, HIV and curative services) will be identified and requested to participate in a semi–structured interview (face to face or telephone).
- *NGO and international organisations* (for example WHO Country office, UNAIDS, PEPFAR, CDC) will be identified and requested to participate in a semi–structured interview (face to face or telephone).

For peer review only - http://bmjopen.bmj.com/site/about/guidelines.xhtml

- *Community leaders* will be identified in the clinic catchment areas by virtue of their position while *community members will be identified* in consultation with the community leaders and invited to participate in a FGD.
- *Clinical researchers* will be invited to participate in an in depth interview at the 24 month end point.

Ethical considerations

Ethical approval for the evaluation has been granted by the research ethics committees of the Liverpool School of Tropical Medicine (UK), the National Institute of Medical Research (Tanzania) and Medical Research Council (Uganda). The key ethical principles of voluntary and informed participation, confidentiality and safety of participants will be used in all researcher and participant interactions. Written consent for interviews and observations will be obtained from all participants. All participants will be provided with written information about the research, this will be explained verbally, and informed that their participation is voluntary and that they may withdraw from participation at any time. Safety and confidentiality of all data will be ensured by: (1) encrypting all transcriptions with a password protected code; (2) storing all data in a secure, encrypted database accessible only to authorised persons on the research team; (3) de-linking all personal information of participants from the data collected and stored and each participant have a unique identification number.

Data analysis and synthesis

The analysis of qualitative data will be iterative, moving between data collection and analysis to test emerging theories. Field notes of observations will be analysed thematically to provide a description of the process and content involved in adapting and delivering the intervention. Audio recordings of interviews and FGDs will be transcribed verbatim by competent and experienced social scientists, with a subsample transcribed using conversation analytic conventions. Translation from local languages (e.g. Swahili, Luganda) into English will be performed for easy sharing with the study partners. Translation will occur using a back translation method for consistency. An electronic data management package (e.g. NVivo) will be used to manage the qualitative data analysis at the respective country levels. The analysis of the observational data will require knowledge obtained from health professional interviews at different levels to compare how reported experience, and different accounts of patient and professional perspectives relate to actual implementation of INTE-AFRICA scenarios: i) when DM and hypertension services are integrated with HIV-infection services; and ii) comparing countries. Care will be taken to identify and follow up deviant cases which do not fit into emerging theories. Reliability and validity of the analysis is optimised through iterative data collection, the use of a multi-method design incorporating interviews, FGD and observations and the ongoing discussion of findings within the research team for scrutiny and feedback. [60,61]

For peer review only - http://bmjopen.bmj.com/site/about/guidelines.xhtml

The chosen phenomenological approach (EPP), usually used in psychological research, reveals the structures of subjective experience and meaning of a lived phenomenon (in first person point of view). It follows to some extent Husserl's principle of active efforts to *''bracket out''* the researchers' theoretical pre-understanding in the first steps of a text analysis [59]. The ''*bracketing*'', however, does not exclude an empathetic, psychological focus in the analysis on the experiences of the researched phenomenon as it is lived by the informant and what it means is to her or him. In the context of INTE-AFRICA, researchers in both countries will strive to get an empathetic understanding of the text, and hence do not apply their professional prior knowledge about integration.

We will conduct a stepwise analysis in five steps. ***First***, the text will be read several times to get a good grasp of how the informant spoke about the researched phenomenon of integration. In this step, theoretical reflection will be withheld. ***Second,*** the whole text will be divided into meaning units of a whole paragraph or a single word. ***Third,*** the informant's personal language will be transformed, unit-by-unit, to the researchers' language. The researchers will discuss the transcription unit by unit. When different interpretations occur, the researchers will return to the interview text and discuss in a free, imaginative process until agreement can be reached through negotiated consensus. ***Fourth***, the text will be screened in a search for comprehensive themes. The text will be interpreted with connection to the researchers' theoretical knowledge in an interchange between the original data, the transformed units and the researchers' theoretical pre-understanding about integration. The meaning units will be assorted into appropriate themes and thus constitute a general structure of the phenomenon of integrated care. ***Fifth,*** this essential structure will penetrate all the revealed themes and thus the meaning of the researched phenomenon of integrated care to the informant.

We will add a further layer of triangulation of perspectives across stakeholders and across conditions (HIV/hypertension/DM/multi/co-morbidity) when raising the abstraction level. Triangulation of socio-behavioural qualitative and observational data, during analysis will occur in order to understand how different types of evidence enhance the overall interpretation of how INTE-AFRICA was implemented, and what the additional health economic and clinical data are, drawing case comparisons across clinics, and across countries, and developing possible explanations for implementation variation. This approach will help to identify factors which are plausibly and/or consistently related to successful or unsuccessful delivery of intervention components. Emerging theories and the relationship of the data to the conceptual literature underpinning the intervention will be discussed and refined at INTE-AFRICA research team meetings throughout the project. The analysis of the observational data will also require knowledge from health professional interviews to compare how reported experience relates to actual implementation of integration at the clinic level.

For peer review only - http://bmjopen.bmj.com/site/about/guidelines.xhtml

Beyond the EPP analysis of triangulated stakeholder narratives, the public understanding of science (PUS) theoretical framework[62] will guide overall qualitative data collection and analysis during INTE-AFRICA. It will unpack how patients and their communities in Uganda and Tanzania understand and use different knowledge on HIV and NCDs in their lives by understanding how they create meaning from scientific findings relating to NCDs and HIV and if, how, and to what degree they incorporate these findings into their everyday lives. This theoretical framework has the capacity to shift public attitudes by connecting and communicating development of innovative scientific concepts in the medical field (in this instance integration of HIV/NCD services in Tanzania and Uganda) to the non-scientific public, and thereby enhance education, training cascade, health policy and practice, and ultimately public understanding of multi-morbidities and sustainable routes to care. Further, it will create a platform for the sharing of lessons learnt, best practices and context adaptation of the final integrated model of care in other African countries (clinical care policies and practice, staff cascade of training, service user education and community awareness raising).

Conclusion

This paper reports the design and methods for the planned process evaluation of the INTE-AFRICA cluster randomised, controlled trial in selected clinics in Tanzania and Uganda. It provides a unique opportunity to document implementation and collaboratively refine integrated care in two sub-Saharan African countries. The process evaluation protocol adheres to recommendations intended to facilitate the standardisation of process evaluation design and reporting [51]. This makes possible the synthesis of results of similar studies elsewhere in the SSA region in future.

Contributorship Statement

All authors contributed to the conceptualisation of the research and contributed to writing the manuscript.

MCVH, MB, JVL, SJ designed the process evaluation protocol.

SJ, MN, SM, JB led the development of the INTE-AFRICA trial.

MCVH drafted the manuscript and all co-authors edited and commented on subsequent drafts. All authors approved the final draft for submission. All authors agree to be accountable for all aspects of the work in ensuring that questions related to the accuracy or integrity of any part of the work are appropriately investigated and resolved.

Funding Statement

The INTE-AFRICA project has received funding from the European Union's Horizon 2020 research and innovation programme under grant agreement No 825698. JVL is supported by a

For peer review only - http://bmjopen.bmj.com/site/about/guidelines.xhtml

For peer review only

Spanish Ministry of Science, Innovation and Universities Miguel Servet grant (Instituto de Salud Carlos III/ESF, European Union [CP18/00074]) and further acknowledges support from the Spanish Ministry of Science, Innovation and Universities through the "Centro de Excelencia Severo Ochoa 2019-2023" Programme (CEX2018-000806-S), and from the Government of Catalonia through the CERCA Programme.

Ethical approval statement

Ethical approval for the evaluation has been granted in 2019 at the Liverpool School of Tropical Medicine (UK), the National Institute of Medical Research (Tanzania) and Medical Research Council (Uganda).

Data sharing statement

No additional data available.

Competing interests statement

None

Provenance and peer review

Not commissioned; internally peer reviewed.

Open access

This is an open access article distributed in accordance with the Creative Commons Attribution 4.0 Unported (CC BY 4.0) license, which permits others to copy, redistribute, remix, transform and build upon this work for any purpose, provided the original work is properly cited, a link to the licence is given, and indication of whether changes were made. See: https:// creative commons. org/ licenses/ by/ 4. 0/.

For peer review only

For peer review only - http://bmjopen.bmj.com/site/about/guidelines.xhtml

References

1. Lim SS, Vos T, Flaxman AD, et al. A comparative risk assessment of burden of disease and injury attributable to 67 risk factors and risk factor clusters in 21 regions, 1990-2010: a systematic analysis for the Global Burden of Disease Study 2010. *Lancet* 2012;380(9859):2224-60.
2. World Health Organization. A global brief on hypertension : silent killer, global public health crisis. Geneva, Switzerland, 2013.
3. Global, regional, and national age-sex specific all-cause and cause-specific mortality for 240 causes of death, 1990-2013: a systematic analysis for the Global Burden of Disease Study 2013. *Lancet* 2015;385(9963):117-71.
4. World Health Organization. Global report on diabetes. Geneva: World Health Organization 2016.
5. Noncommunicable Diseases Progress Monitor, 2017. Geneva: World Health Organization 2017.
6. International Diabetes Foundation. Diabetes Atlas. 7th ed. Brussels: International Diabetes Federation 2015.
7. Xu Y, Chen X, Wang K. Global prevalence of hypertension among people living with HIV: a systematic review and meta-analysis. *J Am Soc Hypertens* 2017;11(8):530-40.
8. Adeloye D, Basquill C. Estimating the prevalence and awareness rates of hypertension in Africa: a systematic analysis. *PLoS One* 2014;9(8):e104300.
9. Atun R, Davies JI, Gale EAM, et al. Diabetes in sub-Saharan Africa: from clinical care to health policy. *Lancet Diabetes Endocrinol* 2017;5(8):622-67.
10. Remais JV, Zeng G, Li G, et al. Convergence of non-communicable and infectious diseases in low- and middle-income countries. *Int J Epidemiol* 2013;42(1):221-7.
11. Ford N, Ball A, Baggaley R, et al. The WHO public health approach to HIV treatment and care: looking back and looking ahead. *Lancet Infect Dis* 2018;18(3):e76-e86.
12. Global, regional, and national comparative risk assessment of 79 behavioural, environmental and occupational, and metabolic risks or clusters of risks, 1990-2015: a systematic analysis for the Global Burden of Disease Study 2015. *Lancet* 2016;388(10053):1659-724.
13. Guwatudde D, Nankya-Mutyoba J, Kalyesubula R, et al. The burden of hypertension in sub-Saharan Africa: a four-country cross sectional study. *BMC Public Health* 2015;15:1211.
14. Sicree R, Shaw J, Zimmet P. Diabetes Atlas International Diabetes Federation: Belgium: International Diabetes Federation, 2006.
15. Addo J, Smeeth L, Leon DA. Hypertension in sub-saharan Africa: a systematic review. *Hypertension* 2007;50(6):1012-8.
16. Chang AY, Gómez-Olivé FX, Manne-Goehler J, et al. Multimorbidity and care for hypertension, diabetes and HIV among older adults in rural South Africa. *Bull World Health Organ* 2019;97(1):10-23.
17. George S, McGrath N, Oni T. The association between a detectable HIV viral load and non-communicable diseases comorbidity in HIV positive adults on antiretroviral therapy in Western Cape, South Africa. *BMC Infect Dis* 2019;19(1):348.
18. Jaffar S, Gill G. The crisis of diabetes in sub-Saharan Africa. *Lancet Diabetes Endocrinol* 2017;5(8):574-75.
19. Jaffar S. Diabetes and other non-communicable diseases in Africa: a potential disaster in the waiting. *Lancet Diabetes Endocrinol* 2016;4(11):875-77.

For peer review only - http://bmjopen.bmj.com/site/about/guidelines.xhtml

20. Lupafya PC, Mwagomba BL, Hosig K, et al. Implementation of Policies and Strategies for Control of Noncommunicable Diseases in Malawi: Challenges and Opportunities. *Health Educ Behav* 2016;43(1 Suppl):64s-9s.
21. Katende D, Mutungi G, Baisley K, et al. Readiness of Ugandan health services for the management of outpatients with chronic diseases. *Trop Med Int Health* 2015;20(10):1385-95.
22. Manne-Goehler J, Atun R, Stokes A, et al. Diabetes diagnosis and care in sub-Saharan Africa: pooled analysis of individual data from 12 countries. *Lancet Diabetes Endocrinol* 2016;4(11):903-12.
23. Peck R, Mghamba J, Vanobberghen F, et al. Preparedness of Tanzanian health facilities for outpatient primary care of hypertension and diabetes: a cross-sectional survey. *Lancet Glob Health* 2014;2(5):e285-92.
24. Price AJ, Crampin AC, Amberbir A, et al. Prevalence of obesity, hypertension, and diabetes, and cascade of care in sub-Saharan Africa: a cross-sectional, population-based study in rural and urban Malawi. *Lancet Diabetes Endocrinol* 2018;6(3):208-22.
25. Patel P, Rose CE, Collins PY, et al. Noncommunicable diseases among HIV-infected persons in low-income and middle-income countries: a systematic review and meta-analysis. *Aids* 2018;32 Suppl 1(Suppl 1):S5-s20.
26. Haregu TN, Setswe G, Elliott J, et al. Integration of HIV/AIDS and noncommunicable diseases in developing countries: rationale, policies and models. *International Journal of Healthcare* 2015;1:21-27.
27. Husain NE, Noor SK, Elmadhoun WM, et al. Diabetes, metabolic syndrome and dyslipidemia in people living with HIV in Africa: re-emerging challenges not to be forgotten. *HIV AIDS (Auckl)* 2017;9:193-202.
28. Juma K, Reid M, Roy M, et al. From HIV prevention to non-communicable disease health promotion efforts in sub-Saharan Africa: A Narrative Review. *Aids* 2018;32 Suppl 1:S63-s73.
29. Rabkin M, Melaku Z, Bruce K, et al. Strengthening Health Systems for Chronic Care: Leveraging HIV Programs to Support Diabetes Services in Ethiopia and Swaziland. *J Trop Med* 2012;2012:137460.
30. Some D, Edwards JK, Reid T, et al. Task Shifting the Management of Non-Communicable Diseases to Nurses in Kibera, Kenya: Does It Work? *PLoS One* 2016;11(1):e0145634.
31. Haldane V, Legido-Quigley H, Chuah FLH, et al. Integrating cardiovascular diseases, hypertension, and diabetes with HIV services: a systematic review. *AIDS Care* 2018;30(1):103-15.
32. Craig P, Dieppe P, Macintyre S, et al. Developing and evaluating complex interventions: the new Medical Research Council guidance. *Bmj* 2008;337:a1655.
33. Iwelunmor J, Ezechi O, Obiezu-Umeh C, et al. Capabilities, opportunities and motivations for integrating evidence-based strategy for hypertension control into HIV clinics in Southwest Nigeria. *PLoS One* 2019;14(6):e0217703.
34. Rachlis B, Naanyu V, Wachira J, et al. Identifying common barriers and facilitators to linkage and retention in chronic disease care in western Kenya. *BMC Public Health* 2016;16:741.
35. Ameh S, Klipstein-Grobusch K, Musenge E, et al. Effectiveness of an Integrated Approach to HIV and Hypertension Care in Rural South Africa: Controlled Interrupted Time-Series Analysis. *J Acquir Immune Defic Syndr* 2017;75(4):472-79.
36. Anand TN, Joseph LM, Geetha AV, et al. Task sharing with non-physician health-care workers for management of blood pressure in low-income and middle-income

For peer review only - http://bmjopen.bmj.com/site/about/guidelines.xhtml

countries: a systematic review and meta-analysis. *Lancet Glob Health* 2019;7(6):e761-e71.
37. Divala OH, Amberbir A, Ismail Z, et al. The burden of hypertension, diabetes mellitus, and cardiovascular risk factors among adult Malawians in HIV care: consequences for integrated services. *BMC Public Health* 2016;16(1):1243.
38. Edwards JK, Bygrave H, Van den Bergh R, et al. HIV with non-communicable diseases in primary care in Kibera, Nairobi, Kenya: characteristics and outcomes 2010-2013. *Trans R Soc Trop Med Hyg* 2015;109(7):440-6.
39. Golovaty I, Sharma M, Van Heerden A, et al. Cost of Integrating Noncommunicable Disease Screening Into Home-Based HIV Testing and Counseling in South Africa. *J Acquir Immune Defic Syndr* 2018;78(5):522-26.
40. Kwarisiima D, Atukunda M, Owaraganise A, et al. Hypertension control in integrated HIV and chronic disease clinics in Uganda in the SEARCH study. *BMC Public Health* 2019;19(1):511.
41. Fairall LR, Folb N, Timmerman V, et al. Educational Outreach with an Integrated Clinical Tool for Nurse-Led Non-communicable Chronic Disease Management in Primary Care in South Africa: A Pragmatic Cluster Randomised Controlled Trial. *PLoS Med* 2016;13(11):e1002178.
42. Mahomed OH, Asmall S. Development and implementation of an integrated chronic disease model in South Africa: lessons in the management of change through improving the quality of clinical practice. *Int J Integr Care* 2015;15:e038.
43. Matanje Mwagomba BL, Ameh S, Bongomin P, et al. Opportunities and challenges for evidence-informed HIV-noncommunicable disease integrated care policies and programs: lessons from Malawi, South Africa, Swaziland and Kenya. *Aids* 2018;32 Suppl 1:S21-s32.
44. Nuche-Berenguer B, Kupfer LE. Readiness of Sub-Saharan Africa Healthcare Systems for the New Pandemic, Diabetes: A Systematic Review. *J Diabetes Res* 2018;2018:9262395.
45. Pfaff C, Scott V, Hoffman R, et al. You can treat my HIV - But can you treat my blood pressure? Availability of integrated HIV and non-communicable disease care in northern Malawi. *Afr J Prim Health Care Fam Med* 2017;9(1):e1-e8.
46. Pfaff C, Singano V, Akello H, et al. Early experiences integrating hypertension and diabetes screening and treatment in a human immunodeficiency virus clinic in Malawi. *Int Health* 2018;10(6):495-501.
47. Rawat A, Uebel K, Moore D, et al. Integrated HIV-Care Into Primary Health Care Clinics and the Influence on Diabetes and Hypertension Care: An Interrupted Time Series Analysis in Free State, South Africa Over 4 Years. *J Acquir Immune Defic Syndr* 2018;77(5):476-83.
48. Jaffar S, Amuron B, Foster S, et al. Rates of virological failure in patients treated in a home-based versus a facility-based HIV-care model in Jinja, southeast Uganda: a cluster-randomised equivalence trial. *Lancet* 2009;374(9707):2080-89.
49. Manne-Goehler J, Atun R, Stokes A, et al. Diabetes diagnosis and care in sub-Saharan Africa: pooled analysis of individual data from 12 countries. *Lancet Diabetes Endocrinol* 2016;4(11):903-12. doi: 10.1016/S2213-8587(16)30181-4 [published Online First: 2016/10/30]
50. Georgeu D, Colvin CJ, Lewin S, et al. Implementing nurse-initiated and managed antiretroviral treatment (NIMART) in South Africa: a qualitative process evaluation of the STRETCH trial. *Implement Sci* 2012;7:66.

For peer review only

For peer review only - http://bmjopen.bmj.com/site/about/guidelines.xhtml

51. Moore G, Audrey S, Barker M, et al. Process evaluation in complex public health intervention studies: the need for guidance. *J Epidemiol Community Health* 2014;68(2):101-2.
52. Linnan L, Steckler A. Process evaluation for public health interventions and research. An overview. In: Steckler A, Linnan L, eds. Process evaluation for public health interventions and research. 1st ed. San Francisco: Josset-Bass 2002:1-23.
53. Murdoch J, Curran R, Bachmann M, et al. Strengthening the quality of paediatric primary care: protocol for the process evaluation of a health systems intervention in South Africa. *BMJ Glob Health* 2018;3(Suppl 5):e000945.
54. Kippax S. Effective HIV prevention: the indispensable role of social science. *J Int AIDS Soc* 2012;15(2):17357.
55. Bronfenbrenner U. Toward an experimental ecology of human development. *American Psychologist* 1977;32(7):513-31.
56. Bronfenbrenner U. The ecology of human development: Experiments by nature and design. Cambridge, MA: Harvard University Press 1979.
57. Hawe P, Shiell A, Riley T. Theorising interventions as events in systems. *Am J Community Psychol* 2009;43(3-4):267-76.
58. Grimshaw JM, Zwarenstein M, Tetroe JM, et al. Looking inside the black box: a theory-based process evaluation alongside a randomised controlled trial of printed educational materials (the Ontario printed educational message, OPEM) to improve referral and prescribing practices in primary care in Ontario, Canada. *Implement Sci* 2007;2:38.
59. Karlsson G. Psychological Qualitative Research From a Phenomenological Perspective: Almqvist & Wiksell International 1993.
60. Marshall C, Rossman GB. Designing qualitative research. 4th ed. Thousands Oaks, Calif: Sage Publications 2006.
61. Patton MQ, Patton MQ. Qualitative research and evaluation methods. Thousand Oaks, Calif.: Sage Publications 2002.
62. Brossard, D and Lewenstein, B (2010). A Critical Appraisal of Models of Public Understanding of Science: Using Practice to inform Theory. Routledge.

For peer review only - http://bmjopen.bmj.com/site/about/guidelines.xhtml

Table One 'Key data on the country settings'

	Tanzania	Uganda
Income level	Low	Low
Population size	58m (2018)	35m (2016)
Estimated prevalence of hypertension from STEPS survey	26%	26%
Estimated prevalence of diabetes from STEPS survey [49] *	5-10%	2-5%
Estimated prevalence of HIV-infection	5.1% (2017)	6.2% (2017)
Doctors density /100,000 population	3 (2014)	0.8 (2005)

* diabetes estimate varies according to age and gender. Data are of variable quality but reference 11 shows that the overall median diabetes prevalence in 12 countries in Africa is 5%.

For peer review only

Table Two 'Logic Model of programme inputs, processes and outcomes'

<table>
<tr><th>Intervention inputs</th><th>Changes in care processes</th><th>Outcomes</th></tr>
<tr><td>Negotiation with national, district and local government health departments, NGOs and funders</td><td>Agreement about and support for service model, including reorganisation of clinics and staff
Commitment to ensure drug supply</td><td>• Reorganisation of clinics and staff to implement the model
• An effective, quality, and sustainable funded drug supply chain</td></tr>
<tr><td>Negotiating lower drug prices
Supporting and monitoring drug ordering in each clinic
Providing buffer drug supplies to each clinic</td><td>Drugs always in stock</td><td rowspan="6">• Increased diagnosis of comorbid conditions
• Increased retention and adherence
• Increased viral suppression, better control of blood pressure and blood glucose
• Less AIDS, cardiovascular disease and diabetes complications
• Lower patients costs (travel and absence from work)
• Less health service duplication and costs (health service costs might increase if more patients are diagnosed and are more adherent)
• Increase patient satisfaction
• Increased clinician satisfaction; less burn-out and absenteeism
• Reduce missed opportunities for improving care and health outcomes</td></tr>
<tr><td>Engagement with and support for clinicians and managers in each clinic</td><td>Clinicians and managers enable and support integration, and find solutions to emerging problems</td></tr>
<tr><td>Provision of integrated service in each clinic (alongside and additional to existing services)</td><td>Trial participants attend integrated service
Avoid multiple visits for patients with multi-morbidity</td></tr>
<tr><td>Training clinicians about integrated clinical management</td><td>Better diagnosis and treatment including attention to comorbid conditions</td></tr>
<tr><td>Community engagement</td><td>Identify and enlist community organisations and resources to help with health education, tracing defaulters or patients who have difficulty attending clinic</td></tr>
<tr><td>Providing standardised stationery for integrated medical records; training clinicians to use it</td><td>Increased awareness by clinicians and patients about disease severity, comorbidity, adherence and control in individual patients</td></tr>
<tr><td>Improving monitoring and evaluation based on clinics registers and medical records</td><td>Regular data analysis and feedback to staff</td><td>Quality assurance and continuous improvement in the quality of care</td></tr>
<tr><td>Identifying effective health education</td><td>Improve health education at clinics</td><td>Healthier lifestyles
Increased adherence</td></tr>
</table>

For peer review only

Table Three: 'Process Evaluation Design and data collection framework'

Post Integration<br>* Numbers indicated are per country/.<br>Data Collection at each Site	6 months	12 months	18 months	Contextual level
Observations of consultations, different processes and clinic flow at clinic levels and in non-clinical areas.	1 week	1 week	1 week	**Micro context (facility and neighbourhoods)**
In depth phenomenological interviews with service users	25	25	25	**Micro context (facility and neighbourhoods)**
In depth phenomenological interviews with health care providers at the clinic (health facility managers, and health professionals such as physicians, nurses, public health nurses)	10	10	10	**Meso context (national to regional/city)**<br>**Micro context (facility and neighbourhoods)**
Semi-structured interviews with Ministerial policy makers and provincial/regional/district level clinical/health senior management (Director for NCD, HIV and curative services).	-	5	5	**Macro context (Global)**<br>**Meso context (national to regional/city)**
Semi-structured interviews with NGO and international organisations (for example WHO Country office, UNAIDS, PEPFAR, CDC)	-	5	5	**Macro context (Global)**<br>**Meso context (national to regional/city)**
Focus group discussions (FGD) with community leaders (8-12 participants)	1	1	1	**Micro context (facility and neighbourhoods)**
Focus group discussions (FGD) gender specific with community members(8-12participants)	2	2	2	**Micro context (facility and neighbourhoods)**
In depth phenomenological interviews with clinical researchers			4	**Micro context (facility and neighbourhoods)**<br>**Meso context (national to regional/city)**

For peer review only

Figure One 'Potential benefits of integrating diabetes, hypertension and HIV services for a) DM and hypertension control, and b) HIV control'

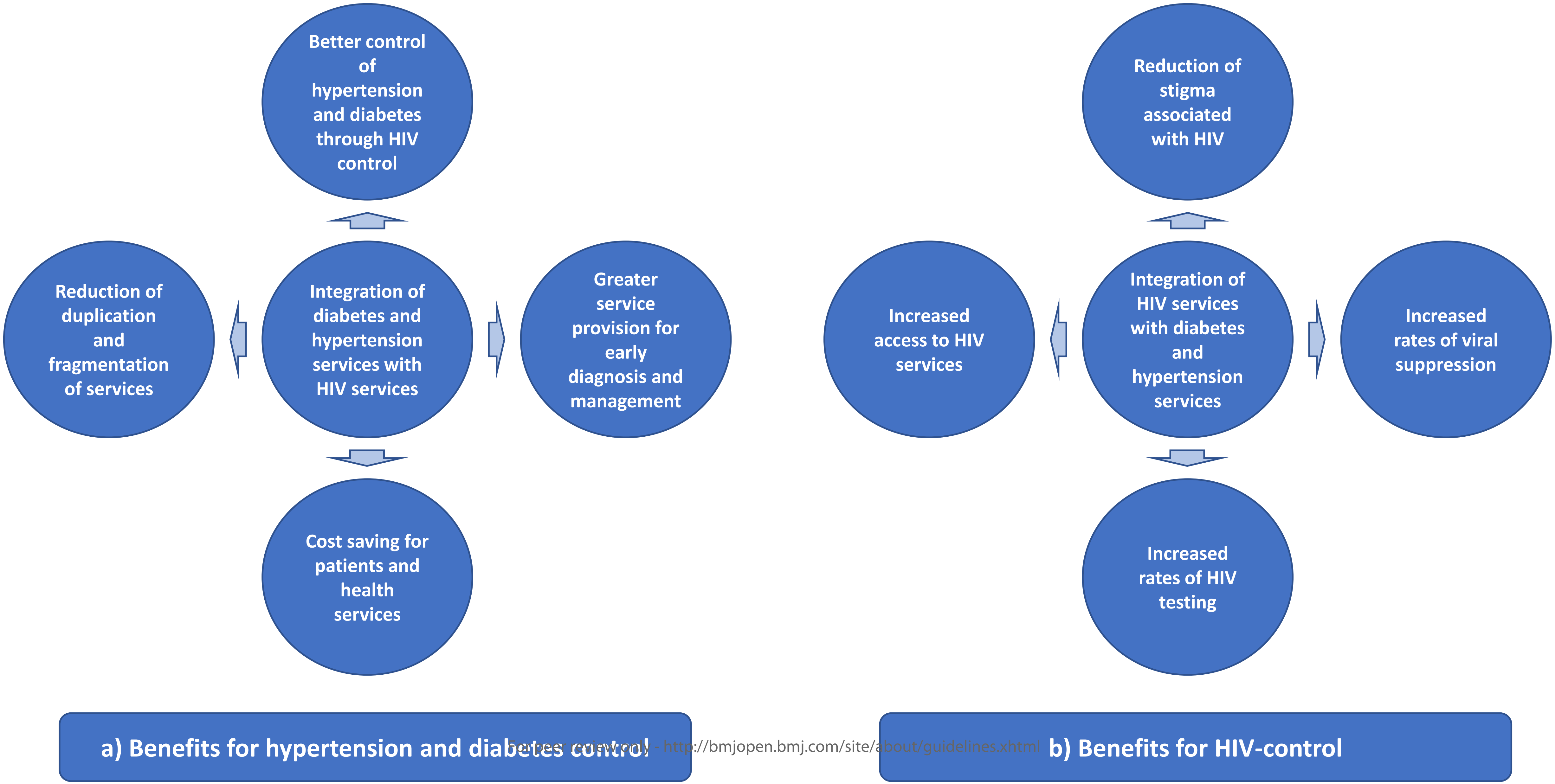

For peer review only - http://bmjopen.bmj.com/site/about/guidelines.xhtml

Figure Two 'INTEAFRICA Conceptual Model'

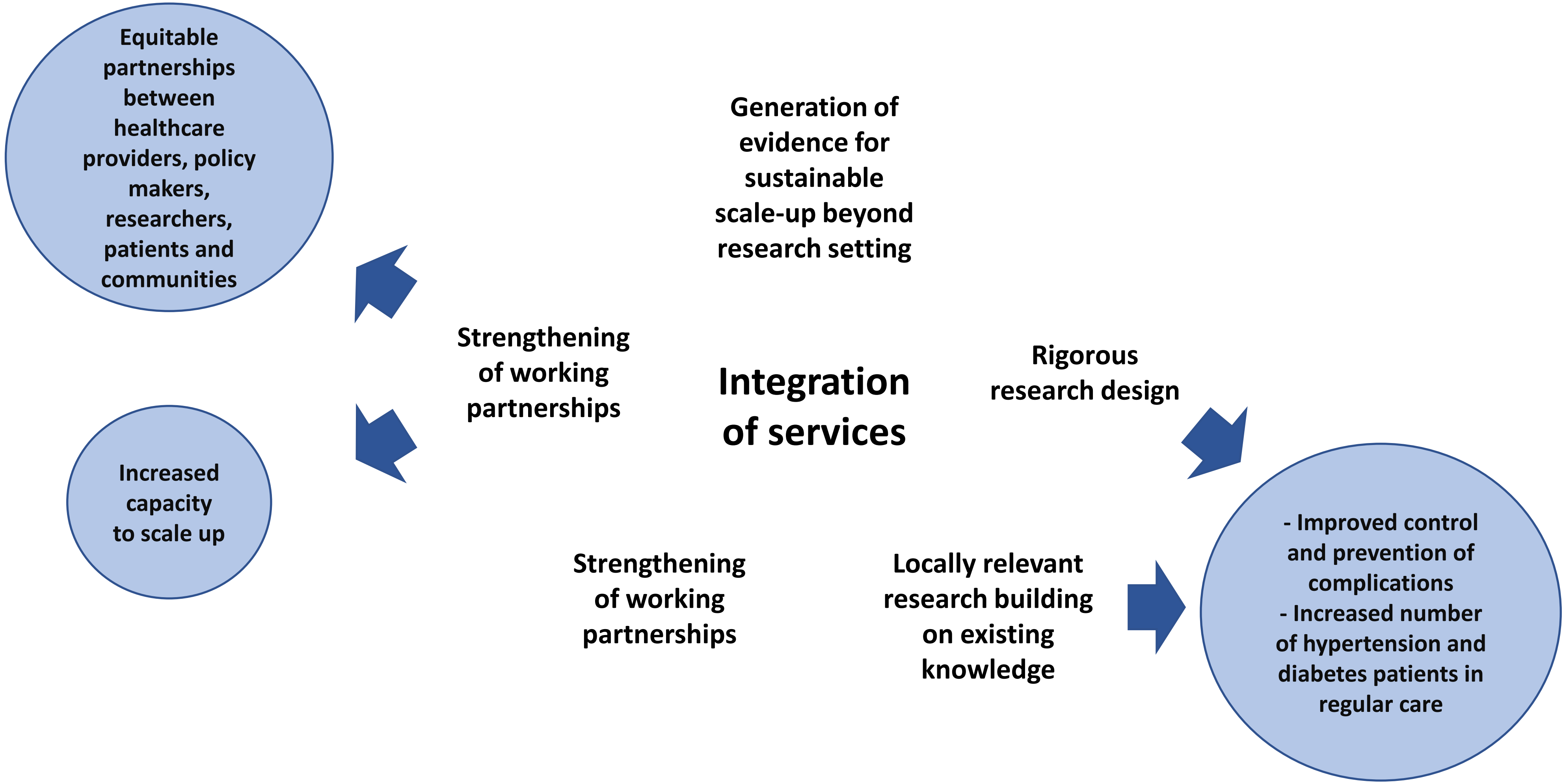

Figure Three 'Potential contextual influences on INTEAFRICA programme implementation cascade'

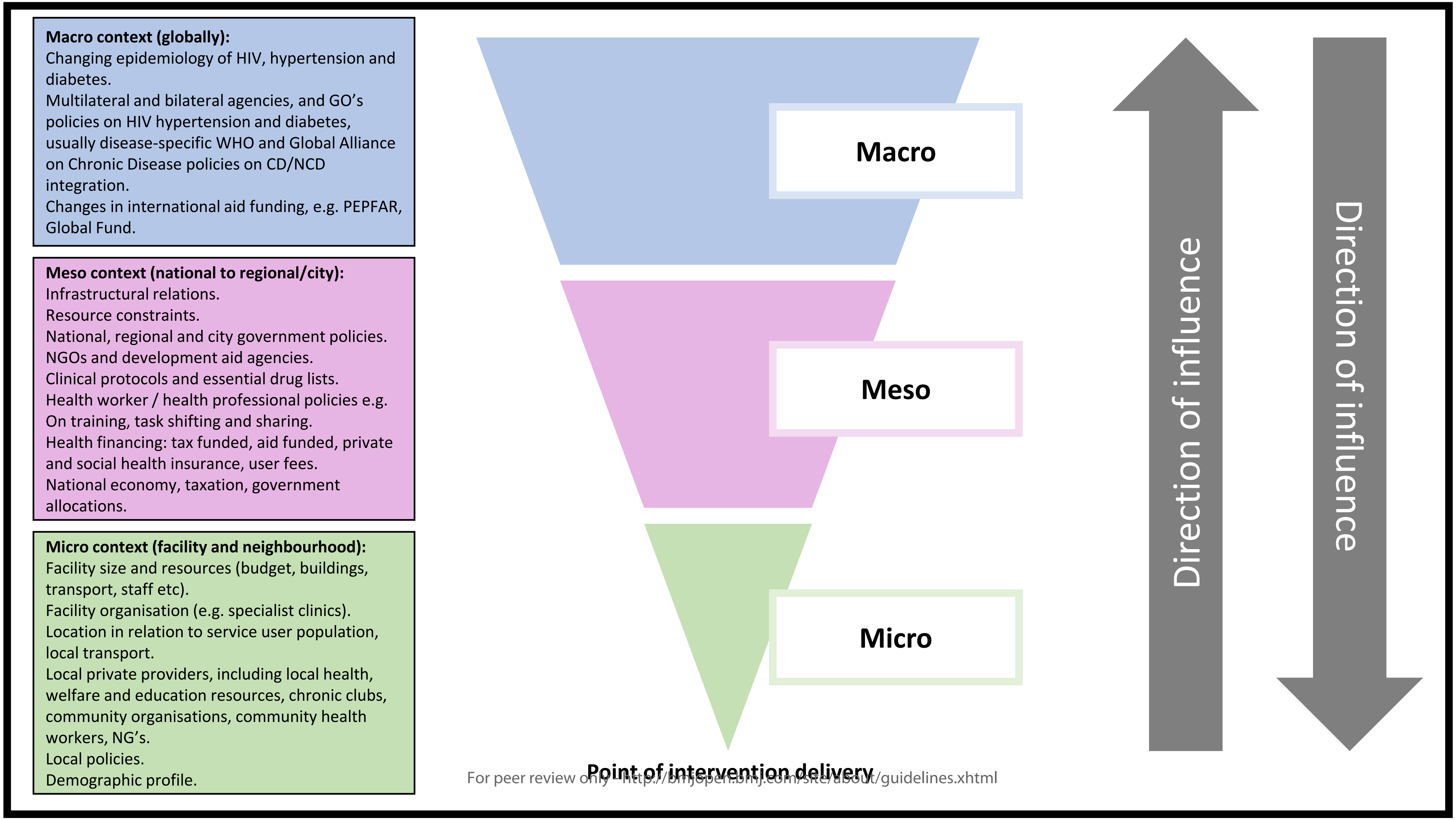

For peer review only - http://bmjopen.bmj.com/site/about/guidelines.xhtml

Strengthening integration of chronic care in Africa: protocol for the qualitative process evaluation of integrated HIV, diabetes and hypertension care in a cluster randomised controlled trial in Uganda and Tanzania.

Journal:	*BMJ Open*
Manuscript ID	bmjopen-2020-039237.R1
Article Type:	Protocol
Date Submitted by the Author:	22-Jul-2020
Complete List of Authors:	Van Hout, Marie-Claire; Liverpool John Moores University, Public Health Institute<br>Bachmann, Max; University of East Anglia Faculty of Medicine and Health Sciences, Norwich Medical School<br>Lazarus, Jeffrey; Instituto de Salud Global Barcelona, Hospital Cliínic, University of Barcelona;<br>Shayo, Elizabeth; National Institute for Medical Research, Muhimbili Centre<br>Bukenya, Dominic; Medical Research Council Uganda, MRC/UVRI/LSHTM Uganda Research Unit<br>Picchio, Camila; Instituto de Salud Global Barcelona, Hospital Clinic University of Barcelona<br>Nyirenda, Moffat; Medical Research Council Uganda , MRC/UVRI/LSHTM Uganda Research Unit<br>Mfinanga, Sayoki; National Institute for Medical Research, Muhimbili Centre<br>Birungi, Josephine; Medical Research Council Uganda , MRC/UVRI/LSHTM Uganda Research Unit<br>Okebe, Joseph; Liverpool School of Tropical Medicine, Department of International Public Health<br>Jaffar, Shabbar; Liverpool School of Tropical Medicine, Department of International Public Health
**Primary Subject Heading**:	HIV/AIDS
Secondary Subject Heading:	Global health, Health services research, Patient-centred medicine, Diabetes and endocrinology
Keywords:	Hypertension < CARDIOLOGY, DIABETES & ENDOCRINOLOGY, International health services < HEALTH SERVICES ADMINISTRATION & MANAGEMENT, Organisation of health services < HEALTH SERVICES ADMINISTRATION & MANAGEMENT

For peer review only - http://bmjopen.bmj.com/site/about/guidelines.xhtml

SCHOLARONE™
Manuscripts

I, the Submitting Author has the right to grant and does grant on behalf of all authors of the Work (as defined in the below author licence), an exclusive licence and/or a non-exclusive licence for contributions from authors who are: i) UK Crown employees; ii) where BMJ has agreed a CC-BY licence shall apply, and/or iii) in accordance with the terms applicable for US Federal Government officers or employees acting as part of their official duties; on a worldwide, perpetual, irrevocable, royalty-free basis to BMJ Publishing Group Ltd ("BMJ") its licensees and where the relevant Journal is co-owned by BMJ to the co-owners of the Journal, to publish the Work in this journal and any other BMJ products and to exploit all rights, as set out in our licence.

The Submitting Author accepts and understands that any supply made under these terms is made by BMJ to the Submitting Author unless you are acting as an employee on behalf of your employer or a postgraduate student of an affiliated institution which is paying any applicable article publishing charge ("APC") for Open Access articles. Where the Submitting Author wishes to make the Work available on an Open Access basis (and intends to pay the relevant APC), the terms of reuse of such Open Access shall be governed by a Creative Commons licence – details of these licences and which Creative Commons licence will apply to this Work are set out in our licence referred to above.

Other than as permitted in any relevant BMJ Author's Self Archiving Policies, I confirm this Work has not been accepted for publication elsewhere, is not being considered for publication elsewhere and does not duplicate material already published. I confirm all authors consent to publication of this Work and authorise the granting of this licence.

For peer review only

Title

Strengthening integration of chronic care in Africa: protocol for the qualitative process evaluation of integrated HIV, diabetes and hypertension care in a cluster randomised controlled trial in Uganda and Tanzania

Author Listing

Marie Claire Van Hout, Liverpool John Moores University, Public Health Institute, United Kingdom

Max Bachmann, University of East Anglia, Faculty of Medicine and Health Sciences, United Kingdom

Jeffrey V Lazarus, Instituto de Salud Global Barcelona (Barcelona Institute for Global Health ISGlobal), Hospital Clínic, University of Barcelona, Spain

Elizabeth H. Shayo, National Institute for Medical Research (NIMR), Muhimbili Centre, Tanzania

Dominic Bukenya, Medical Research Council, MRC/UVRI/LSHTM Uganda Research Unit, Uganda

Camila A Picchio, Instituto de Salud Global Barcelona (Barcelona Institute for Global Health ISGlobal), Hospital Clínic, University of Barcelona, Spain

Moffat Nyirenda, Medical Research Council, MRC/UVRI/LSHTM Uganda Research Unit, Uganda

Sayoki Mfinanga, National Institute for Medical Research (NIMR), Muhimbili Centre, Tanzania

Josephine Birungi, Medical Research Council, MRC/UVRI/LSHTM Uganda Research Unit, Uganda

Joseph Okebe, Liverpool School of Tropical Medicine, Department of International Public Health, United Kingdom

Shabbar Jaffar, Liverpool School of Tropical Medicine, Department of International Public Health, United Kingdom

Corresponding Author:

Professor Marie Claire Van Hout, Public Health Institute, Exchange Station, Liverpool John Moores University, Liverpool L32ET, United Kingdom. Email: m.c.vanhout@ljmu.ac.uk

Article Type: Protocol

For peer review only - http://bmjopen.bmj.com/site/about/guidelines.xhtml

For peer review only

Abstract

Introduction In sub-Saharan Africa (SSA), the burden of non-communicable diseases (NCDs), particularly diabetes mellitus (DM) and hypertension, has increased rapidly in recent years, although HIV infections remain a leading cause of death among young-middle aged adults. Health service coverage for NCDs remains very low in contrast to HIV, despite the increasing prevalence of co-morbidity of NCDs with HIV. There is an urgent need to expand healthcare capacity to provide integrated services to address these chronic conditions.

Methods and Analysis: This protocol describes procedures for a qualitative process evaluation of the INTE-AFRICA, a cluster-randomised trial comparing integrated health service provision for HIV-infection, DM, and hypertension, to the current stand-alone vertical care. Interviews, focus group discussions, and observations of consultations and other care processes in two clinics (Tanzania, Uganda) will be used to explore the experiences of stakeholders. These stakeholders will include health service users, policymakers, healthcare providers, community leaders and members, researchers, non-governmental (NGO) and international organisations. The exploration will be carried out during the implementation of the project, alongside an understanding of the impact of broader structural and contextual factors.

Ethics and Dissemination: Ethical approval has been granted by Liverpool School of Tropical Medicine (UK), the National Institute of Medical Research (Tanzania) and TASO Research Ethics Committee (Uganda). The evaluation will provide the opportunity to document the implementation of integration over several time points (six, 12 and 18 months) and refine integrated service provision prior to scale-up. This synergistic approach to evaluate, understand and respond will support service integration and inform monitoring, policy and practice development efforts to involve and educate communities in Uganda and Tanzania. It will create a model of care and a platform of best practices and lessons learnt for other countries implementing integrated and decentralised community health services.

Key Words

Health service delivery, Non communicable disease, HIV, integration, task-shifting, sub-Saharan Africa

For peer review only

Article Summary: Strengths and Limitations of this study

- The INTE-AFRICA trial will implement integration of HIV/NCD services in Tanzania and Uganda in response to an urgent need to respond to increased burden of NCDs, and expand capacity of healthcare systems to manage co-morbidity.
- The INTE-AFRICA trial is based on a partnership between African and European researchers, working closely with policy makers and other stakeholders.
- The process evaluation of INTE-AFRICA uses qualitative and observational methods at two facilities to explore and document stakeholder experiences of integration of services, alongside an understanding of the impact of broader structural and contextual factors.
- The process evaluation works in tandem with quantitative evaluation of clinical efficacy and cost effectiveness of the INTE-AFRICA trial.
- Limitations of the process evaluation may centre on patient drop out, characteristics of the two selected sites and other external mitigating factors.

For peer review only

BACKGROUND

Non-communicable diseases (NCDs) have risen rapidly in Africa, alongside a continuing high burden of HIV-infection. In the sub-Saharan Africa (SSA) region, HIV/AIDS remains a major cause of morbidity and mortality among young-middle aged adults, but the region is also experiencing a rapidly rising burden of NCDs (particularly DM and hypertension), giving rise to a dual HIV-NCD epidemic.[1-3] While lifestyle changes associated with urbanisation and globalization (such as eating habits and lack of physical exercise) underpin the ongoing demographic and epidemiologic transition toward increasing NCDs, these changes also affect chronic conditions (such as HIV).[1-3] The number of people in regular HIV care is rising[4], alongside the rate of NCDs in the SSA region, with hypertension representing the single largest risk factor for death, and DM which has seen a massive increase in prevalence in a short period of time.[2 5-7] Patient populations in SSA are increasingly demonstrating younger age of onset of NCDs, with co-morbidity of NCDs with HIV, and the impact of NCDs is particularly severe in populations affected by poverty.[3 8-11]

Since 2003, significant global investment and development partner engagement has facilitated the establishment of HIV screening and treatment programmes as the first large-scale chronic disease initiatives in Africa. Health services for HIV are stand-alone and vertically delivered. They have also been combined with decentralisation and task-shifting, which has enabled primary health centres to treat large numbers of patients. Non-clinically qualified health workers play a major role in supporting patients on HIV treatment, with almost 70% of people living with HIV-infection in regular care. In contrast, health service coverage for NCDs remains very low.[2 12] Coordinated national NCD control and care programmes are relatively new with significant gaps in funding and operational evidence for program implementation.[11 13.] For example, only about 5-10% of persons with DM are thought to be in regular diabetes care, and the figure is likely to be similar for persons with hypertension.[2 8 11] Furthermore, those in care experience insufficient health service provision for the diagnosis and management of DM and hypertension (medicines supply is patchy).[2 12 14-17]. HIV populations at risk of developing NCDs, and presenting with co/multi-morbidities could potentially impact on the gains from the achieved scale-up of HIV care services.[18]

Hence, there is an urgent need to expand the capacity of healthcare systems in the SSA region to provide services for NCDs, either alongside, or integrated, with HIV.[19] As chronic conditions, DM, hypertension and HIV-infection require lifelong care. Key challenges to chronic care in SSA are linkage and retention in care, access and medicines adherence.[20 21] As such, the infrastructure and lessons learnt from the HIV chronic disease model can serve as important resources for the expansion of NCD prevention, care and treatment. HIV chronic care management pathways, resources and infrastructure can be leveraged to integrate with newly developing NCD services, ultimately to strengthen the platform for NCD services and improve health outcomes for people with NCDs.[22-24]

For peer review only - http://bmjopen.bmj.com/site/about/guidelines.xhtml

Health systems have the developed experience managing HIV as a chronic disease, including linking and retaining patients in care and supporting treatment adherence in drugs, diagnostics procurement and other key health systems indicators.[21 25-27] Integration can reduce duplication and fragmentation, streamline services by treat those with co/multi-morbidities, and potentially offer patient benefits relating to time and cost. See **Figure One**. It could however also threaten service capacity by resulting in increased service demand and loss of clinical focus on one disease.

Insert **Figure One 'Potential benefits of integrating diabetes, hypertension and HIV services for a) DM and hypertension control, and b HIV control'** about here

Despite the increase in academic and clinical interest in HIV/NCD integration in SSA, [9 26 28-33] little evidence about integration in terms of both scope and generalizability exists. Extant evidence is limited to small scale feasibility studies in largely different contexts [34-40], despite suggesting that integrated care is an efficient use of resources compared to the standard of care and beneficial for patients' NCD and HIV clinical outcomes.[28 30 32] There is a lack of large scale or randomized and/or controlled evaluations and context-specific clinical, cost effectiveness and process outcomes data constrains policymaking and development of integrated care models, which could strengthen health systems when tailored to the distinct needs of each specific SSA country.[19 21 37] Such evidence is paramount to inform policy, government resource prioritisation, and to develop integrated care models which strengthen health systems best suited to the needs of that specific SSA country. The INTE-AFRICA trial is conducted to respond to the insufficient existing evidence to substantiate the benefits and sustainability of integrated care as well as implementation of such programmes in the SSA setting. We present here the qualitative process evaluation protocol designed to evaluate INTE-AFRICA, a European Commission Horizon 2020 funded implementation research project (protocol number 19-100) operating in Uganda and Tanzania.

For peer review only

The INTE-AFRICA Trial

INTE-AFRICA aims to implement and assess the effectiveness of integration of HIV, DM and hypertension services at the point of service delivery covering many health facilities where common approaches to clinical decision-making, drug procurement and human resource management occur. The project will generate the research evidence needed by health services in Africa, to scale-up and sustain chronic disease management and services in an integrated manner. We are also interested to observe changing dynamics pertaining to stigma and discrimination associated with HIV.[41]

Tanzania and Uganda were specifically chosen by INTE-AFRICA as they are low-income countries and their public and private health facilities are strongly committed to providing services for NCD. However, their health systems struggle to scale-up provision for diabetes and hypertension in the face

For peer review only - http://bmjopen.bmj.com/site/about/guidelines.xhtml

of competing health demands, including HIV-infection. See **Table One**. Tanzania and Uganda also share relevant characteristics with other countries in SSA. As such, the process evaluation has the potential to enhance the generalisability of integrated care to other similar settings by providing understanding of the determinants and mechanisms of the implementation process.

Insert **Table One 'Key data on the country settings'** about here

Our programme is underpinned by a participatory, multi-actor approach which supports dialogue and knowledge exchange, fosters mutual understanding and provides input in policy agendas around diagnosis and treatment of DM, hypertension and HIV in an integrated clinic. The INTE-AFRICA conceptual framework is illustrated in **Figure Two.**

Insert **Figure Two 'INTEAFRICA Conceptual Model'** about here

INTE-AFRICA will test integrated health services at primary care centres for HIV-infection, diabetes and hypertension, by providing a "*One stop*" integrated care clinic for these conditions (the intervention). We will conduct a pragmatic parallel arm cluster-randomised trial: 32 largely urban health facilities offering primary care services in the two countries will be randomised, with 16 facilities allocated to deliver the intervention immediately (intervention arm), and 16 facilities to continue with usual care (control arm). At each selected facility, cohorts of approximately 220 patients; 110 HIV infected and 110 NCD patients, will be enrolled to evaluate the primary research outcomes.

The primary care facilities will initiate and stabilise patients on treatment, manage complications (including referral to higher facilities) and conduct clinical and laboratory monitoring of patients for all three conditions. Specific characteristics of integrated care in each '*One stop*' integrated care clinic are: concurrent management of HIV, hypertension and DM in the same facility; management of patients with HIV, hypertension and DM by the same clinician or team of clinicians (nurses, counsellors other staff); integrated training of clinicians; single waiting area and queue; integrated health education about all three conditions; one pharmacy with a single drug dispensing point; similar testing, and cross-testing, for diagnosis and monitoring with the requisition of laboratory tests in the same place; and similar format of paper medical records for each condition kept in the same patient folder.

Patients who decline to participate in the research and trial participants in control arm clinics will continue to receive standard vertical health care delivery. Inclusion criteria for participation are: being over 18 years old; having confirmed HIV-infection, DM or hypertension; living within the catchment

For peer review only - http://bmjopen.bmj.com/site/about/guidelines.xhtml

population of the health facility; likely to remain in the catchment population for six months and willing to provide written informed consent. Very ill patients requiring in-patient care will be excluded. The research team will observe participants during clinic visits with additional reviews at six and 12 months. Further details are provided in the full INTE-AFRICA trial protocol.

Qualitative Process Evaluation of the INTE AFRICA trial

The aim of the process evaluation of INTE-AFRICA is to explore the experiences, attitudes and practices of a wide variety of stakeholders during the process of programme implementation and to develop an understanding of the impact of broader structural and contextual factors on the implementation process of service integration. Process evaluations typically evaluate how and whether interventions are delivered as intended and whether such implementation is congruent with the theory underpinning the intervention. [25 42-44] Updated Medical Research Council (MRC) guidance for evaluation of complex health interventions has recently recognised the value of process evaluation within trials stating, "*it can be used to assess fidelity and quality of implementation, clarify causal mechanisms and identify contextual factors associated with variation in outcomes.*" [25] Hence, the process evaluation in INTE-AFRICA is particularly focused on context, description of the intervention and its causal assumptions, implementation, mechanisms of impact and outcomes. [25] Further we evaluate the extent to which resources and activities supporting the intervention function to deliver intended outputs, with subsequent improvements in outcomes.

A central focus lies in identifying contextually relevant strategies for successful implementation of service integration, and practical difficulties in adoption, delivery and maintenance to inform wider implementation.[45] We recognise that outcomes (e.g. knowledge gained) of INTE-AFRICA are dependent on understanding cultures and contexts of the stakeholders (i.e. patients, healthcare providers, policy makers, community leaders/members, non-governmental organisations (NGO), international organisations, and clinical researchers) involved and those surrounding service design and delivery as well as care seeking practices.

The role of social and behavioural science approaches to understanding individual experience, and the role of such contextual dynamics is central to this process evaluation. It aims to enhance understanding of issues related to the NCD and HIV agendas and service delivery approaches from all perspectives and stakeholders along the integration process. INTE-AFRICA will utilise a broad social behavioural approach to support community engagement in research in Uganda and Tanzania, going beyond clinical trial recruitment and retention to improve NCD/ HIV literacy. Behavioural and social science research strategies [46] will allow NCD and HIV related research to place the needs and perspectives of people living with NCD and/or HIV at the centre of the progression of clinical studies, such as INTE-AFRICA, and investigate participant motivations and decision-making processes

For peer review only - http://bmjopen.bmj.com/site/about/guidelines.xhtml

related to preferences for or participation in different types of integrated and decentralised services.

Theoretical Framework: Bronfenbrenner's ecological model of behaviour

Process evaluation design, like healthcare interventions, requires a theoretical framework to structure the qualitative and observational evaluation across sites. The chosen theoretical framework is Bronfenbrenner's ecological model of behaviour [47 48] used to conceptualise integrated care as events that disrupt complex social systems[49] operating across multiple contextual levels. We are interested in better understanding the importance of context, which becomes especially relevant when comparing Tanzania and Uganda. For example, healthcare coverage and charging dynamics between countries and between HIV as opposed to NCD care differs. In Tanzania, whilst HIV services and drugs for HIV are free, patients are required to pay a direct user fee (unless elderly or very poor) or use their health insurance to pay for NCD drugs. Currently there is a strategy to progress to single health insurance to support universal health coverage in Tanzania. In Uganda, HIV services are also free to all (public and private not for profit health facilities) and HIV drugs are always in stock. However, in Uganda whilst NCD services are free in public facilities, stockouts of laboratory reagents and NCD drugs are frequent, and patients have to pay a direct user fee to access them. There is also no national insurance scheme in Uganda, only private insurance which is relatively expensive and optional.

Using the Bronfenbrenner theoretical framework, contextual factors are evaluated at the ***macro*** (universal vs partial coverage; free primary healthcare and drugs, affordability and users fees; fiscal policy, financial aspects at government level, funding barriers for chronic care management and barriers to government scale-up of service integration); ***meso*** (HIV/NCD drug ordering, drug delivery systems and continuity of supply, health care provider education and employment, community understanding and perspectives on multi-morbidity); and ***micro*** (clinic and pharmacy level management, resources, patient/service user experience) levels. See **Figure Three.** These contextual factors, likely to influence implementation of integration and their effects, will be investigated to capture variation in adoption, delivery and maintenance outcomes as well as responses to the intervention. This will affect both reach and fidelity, which are hypothesised to be important factors in outcome differences.

Insert **Figure Three 'Potential contextual influences on INTE AFRICA programme implementation cascade'** about here

To situate INTE-AFRICA within the Bronfenbrenner theoretical framework, we will develop a logic model. This model will set out contextual determinants of HIV and NCD care management in SSA

For peer review only - http://bmjopen.bmj.com/site/about/guidelines.xhtml

and assess how integrated care components function to address these determinants to improve outcomes in the management and support of patients. See **Table Two.**

Insert **Table Two 'Logic Model of programme inputs, processes and outcomes'** about here

METHODS

Study Setting

This will be a cohort study taking place in one "*One stop*" integrated care clinic per country. In Tanzania, the selected site is *Temeke Regional Referral Hospital* in Dar Es Salaam, a public tertiary health facility, with 465 staff, and serving a population of over 1 million. In Uganda, the selected site is the *Kasangati Health Centre IV*, Kasangati, a public district health facility located in the Wakiso District serving a population of over 2 million. Both provide a range of services to the community (primary care, HIV/AIDs, NCDs, surgical, maternal and child health, health education, dental and pharmacy).

Study Design

This protocol describes procedures for a qualitative process evaluation of the INTE-AFRICA pragmatic parallel arm cluster-randomised trial comparing integrated health service provision for HIV-infection, DM and hypertension with the current standard vertical care delivery model. The process evaluation works in tandem with the collection of selected clinical outcomes (e.g. clinical efficacy of different treatments) and health economic data (e.g. costs and benefits of different approaches) to estimate the potential benefits to patients and health services at clinic and country level (protocol reported elsewhere).

Patient and Public Involvement

Patient and public involvement (PPI) throughout a programme of research enhances research quality and relevance by providing different perspectives and a sense of ownership. This protocol will adhere to the same principles, and will allow the voice of '*service users*' and those affected to be heard, and utilised. Key stakeholders such as patients as service users and their families will be fully involved in guiding the research, acting as research participants, and in implementation of change in health service delivery and integrated care planning. All aspects of the process evaluation are underpinned by participatory action health research and its success and usefulness will be grounded in PPI, participation and engagement in the form of patient/professional identification of research priorities, collaborations and partnerships, expert steering, community participation around health needs and optimal integrated services, awareness raising activities, development of print materials, toolkits and training for healthcare professionals.

For peer review only - http://bmjopen.bmj.com/site/about/guidelines.xhtml

It will use three qualitative research techniques: in-depth interviews with stakeholders (patients, healthcare provider, policy maker, non-governmental organisation (NGO)/international organisation, and clinical researcher); focus group discussions (FGD) with community leaders and community members; and clinic-based observations in one site per country. This design permits an assessment of the fidelity of INTE-AFRICA's implementation, a detailed description of the processes, relationships, and contexts involved in the delivery of integrated care, and the identification of factors attributing to the failure or success of the programme. It thus addresses the *'black box'* problem in interpreting trial results by improving understanding of the mechanisms that connect particular intervention components to particular outcomes.[50] The chosen approach will enhance social construction and acceptability of chosen decentralised integrated approaches, link outcomes to policy and advocacy and impact sustainability of HIV, DM and hypertension chronic disease service integration in two LMIC countries. It provides the opportunity to document and refine INTE-AFRICA activities prior to a larger pragmatic trial or scale-up by Uganda and Tanzanian governments. These synergistic approaches to evaluate, understand and respond will support integration, support affordability, address barriers to government scale up and funding barriers for chronic care, inform surveillance, policy and practice development and improve efforts to involve and educate communities in Uganda and Tanzania. It will create a model and a platform of best practices and lessons learnt for other countries implementing integrated and decentralised community health services for HIV and chronic disease. The process evaluation methods for each objective are described in **Table Three**, including how each method maps onto the three different contextual levels.

Insert **Table Three: 'Process Evaluation Design and data collection framework'** about here.

Study Population and Recruitment

25 patients and 10 health care providers in *Temeke Regional Referral Hospital* in Dar Es Salaam, Tanzania and *Kasangati Health Centre IV*, Kasangati, Uganda will be purposely selected and invited; at six, 12 and 18 months after the start of the trial, to reflect retrospectively on their experience of integration. Health care providers include the hospital/health centre overall in charge and pharmacist, and the '*One stop'* medical officers in-charge, trained clinicians managing HIV, diabetes and hypertension patients, pharmacist, laboratory technician, counsellors or nurses providing health education and counselling, and nurses in the registration desk who are also responsible in taking vital signs from patients. We will also collect qualitative data from interviews with Ministerial policy makers and provincial/regional/district level clinical/health senior management (directors for NCD, HIV and curative services); NGO and international organisations (for example WHO Country office, UNAIDS, PEPFAR, CDC) and clinical researchers; and conduct FGD with community leaders and community members. These numbers are expected to reach saturation (i.e. the point that further information does not provide any additional variation in observed themes). We may replace

For peer review only - http://bmjopen.bmj.com/site/about/guidelines.xhtml

participants, for example patients if there is significant loss to follow up or refusal for repeated interviews. For instance, if a participant drops out at 12 months, we still have their six-month experience documented, and we can replace with a new participant, invited to reflect on their 12-month retrospective experience. Where possible we will gender match interviewers with participants (particularly patients). Participants (patients, healthcare providers and others) will not be directly compensated bur rather they will be compensated for incurred transport costs to attend the interview/FGD, and provided with refreshments during the interview/FGD.

The following recruitment procedures will take place at each '*One stop*' integrated care clinic;

- Observations will be made in the integrated clinic in consultation with the hospital/health centre and '*One stop*' clinic in-charges.
- Recruitment of patients will be supported by clinic nurses who will identify and approach selected participants who have a minimum of six months experience of integration, and will be asked to consent to partake in an in-depth interview on the day they attend the clinic. INTE-AFRICA researchers will purposively sample women and men of different ages and explore any age/gender and condition ((HIV/hypertensive/DM/multi/co-morbid) related differences.
- *Health care providers* at the integrated clinic will be approached to participate in an in-depth interview on the day the INTE-AFRICA team are scheduled to attend.
- *Ministerial policy makers and provincial/regional/district level clinical/health senior management* will be identified and requested to participate in a semi–structured interview (face to face, online using Zoom or telephone).
- *NGO and international organisations* will be identified and requested to participate in a semi–structured interview (face to face or telephone).
- *Community leaders* will be identified in the clinic catchment areas by virtue of their position while *community members* will be identified in consultation with the community leaders and invited to participate in the FGDs.
- *Clinical researchers* will be invited to participate in an in depth interview at the 24 month end point.

Data collection

We will use the Empirical Phenomenological Psychological (EPP) five-step method,[51] which combines psychological, interpretative and idiographic components, to collect data. The data will garner an understanding of the complex social processes, of social, aged, gendered and culturally/community specific meanings and broaden the incremental understanding of the distinct lived experience of policy makers, patients, health care providers, researchers and communities. We

For peer review only - http://bmjopen.bmj.com/site/about/guidelines.xhtml

will balance the description of phenomena with the interpretation of insights and are cognisant of participant experiential phenomena and authors' interpretation of associated meanings. It will yield an in-depth socio-cultural understanding of patient /participant reported outcomes, their motivations, preferences, beliefs, expectations, identities, hopes and views on conditions, related stigma and of decision-making processes. This will provide better understanding of stakeholder and community positioning during integration. This understanding will inform policy and practice, ensure effective patient/service user education, position service users and their families to understand these conditions and interpret study outcomes and facilitate future HIV and chronic disease clinical studies.

Both descriptive patient level data and rich socio-behavioural qualitative/observational data will be collected by a team of trained researchers in Tanzania and Uganda. Data collection will entail exploring the experiences, attitudes and practices of a wide variety of stakeholders during the process of INTE-AFRICA programme implementation and will develop an understanding of the impact of broader structural and contextual factors on the implementation process. [10 45]We will collect data on social behavioural and cultural aspects impacting implementation (e.g. individual and community health risks, protective behaviours and health responses) within the broader social and political frameworks (government resources and barriers to sustaining integration), the practicalities of accessing, providing and sustaining integrated services (e.g. staff time, resources, equity of access, supply chain dynamics and pharmacy components pertaining to drug types, drug/reagent availability and costs for integrated patients, catchment area/populations, quality of care, waiting room dynamics, record keeping and retention across multi/co-morbidities, training gaps); and process indicators (e.g. perceived stigma, acceptability of vertical versus integrated service designs, lay knowledge and awareness, the dynamics of public versus private sector integration (where relevant to the participant), and bottlenecks to accessing services). We will also describe implementation of the intervention in terms of fidelity to the intended model of care, adaptations to the intervention during implementation, and dose and reach of intervention components actually delivered and received (such as numbers and proportions of eligible staff who received integrated care training, numbers of proportions of patient participants who received all or most of their care from integrated services, and frequency of drug stock-outs). The latter data will be complemented by routinely collected quantitative data such as training attendance and medical records. We will document changes in healthcare provider roles, attitudes and patient relationships.

Interviews with patients/service users, healthcare provider and policymaker/senior manager, will include specific questions about their experience and management of individuals with multi-morbid HIV, hypertension and/or diabetes. These include their perceptions of INTE-AFRICA; impacts of INTE-AFRICA on the provision of integrated HIV/AIDS care and NCD care, and relationships with NGO and international organisations; changes in health provider roles, attitudes, and patient

For peer review only - http://bmjopen.bmj.com/site/about/guidelines.xhtml

relationships; impacts of the INTE-AFRICA implementation context on trial and health economic cost outcomes; impacts of the INTE-AFRICA intervention on an integrated health systems approach to care (medicine supplies, record keeping, service user education, clinical care pathways, data management, staff training); and barriers to and facilitators of change and future sustainability of integrated care provision. We will assume a more pragmatic approach when garnering perspectives from higher level stakeholders involved in health policy and practice generation, and NGO and international organisations (e.g. WHO Country offices, UNAIDS, PEPFAR, CDC) providing peripheral supports and guidance. We are especially interested in better understanding the complexities around government scale up and resource allocation for chronic care (for example decentralisation, financial planning, identification of potential funding sources at ministry levels, subsidised NCD drugs, and by international donors (CDC, PEPFAR, UNAIDS). See **Table Three.**

Data analysis and synthesis

The analysis of qualitative data will be iterative, moving between data collection and analysis to test emerging theories. Field notes of observations will be analysed thematically to provide a description of the process and content involved in adapting and delivering the intervention. Audio recordings of interviews and FGDs will be transcribed verbatim by competent and experienced social scientists, with a subsample transcribed using conversation analytic conventions. Translation from local languages (e.g. Swahili, Luganda) into English will be performed for easy sharing with the study partners. Translation will occur using a back translation method for consistency. An electronic data management package (e.g. NVivo) will be used to manage the qualitative data analysis at the respective country levels. The analysis of the observational data will require knowledge obtained from health professional interviews at different levels to compare how reported experience, and different accounts of patient and professional perspectives relate to actual implementation of INTE-AFRICA scenarios: i) when DM and hypertension services are integrated with HIV-infection services; and ii) comparing countries. Care will be taken to identify and follow up deviant cases which do not fit into emerging theories. Reliability and validity of the analysis is optimised through iterative data collection, the use of a multi-method design incorporating interviews, FGD and observations and the ongoing discussion of findings within the research team for scrutiny and feedback. [52 53]

The chosen phenomenological approach (EPP) to collecting and analysing data, usually used in psychological research, reveals the structures of subjective experience and meaning of a lived phenomenon (in first person point of view). It follows to some extent Husserl's principle of active efforts to *''bracket out''* the researchers' theoretical pre-understanding in the first steps of a text analysis. [51] The ''*bracketing*'', however, does not exclude an empathetic, psychological focus in the analysis on the experiences of the researched phenomenon as it is lived by the informant and what it means is to her or him. In the context of INTE-AFRICA, researchers in both countries will strive to

For peer review only - http://bmjopen.bmj.com/site/about/guidelines.xhtml

get an empathetic understanding of the text, and hence do not apply their professional prior knowledge about integration. The analysis of the observational data will also require knowledge from health professional interviews to compare how reported experience relates to actual implementation of integration at the clinic level.

We will conduct a stepwise EPP analysis in five steps. ***First***, the text will be read several times to get a good grasp of how the informant spoke about the researched phenomenon of integration. In this step, theoretical reflection will be withheld. ***Second,*** the whole text will be divided into meaning units of a whole paragraph or a single word. ***Third,*** the informant's personal language will be transformed, unit-by-unit, to the researchers' language. The researchers will discuss the transcription unit by unit. When different interpretations occur, the researchers will return to the interview text and discuss in a free, imaginative process until agreement can be reached through negotiated consensus. ***Fourth***, the text will be screened in a search for comprehensive themes. The text will be interpreted with connection to the researchers' theoretical knowledge in an interchange between the original data, the transformed units and the researchers' theoretical pre-understanding about integration. The meaning units will be assorted into appropriate themes and thus constitute a general structure of the phenomenon of integrated care. ***Fifth,*** this essential structure will penetrate all the revealed themes and thus the meaning of the researched phenomenon of integrated care to the informant.

Credibility and transferability

The process evaluation protocol adheres to recommendations intended to facilitate the standardisation of process evaluation design and reporting [43]. It provides a unique opportunity to document implementation and collaboratively refine integrated care in two sub-Saharan African countries. This makes possible the synthesis of results of similar studies elsewhere in the SSA region in future. In order to ensure credibility, whilst we utilise different methods of data collection (qualitative/observational), and operate concurrently with clinical outcomes data and health economics analysis, we will also add a further layer of triangulation of sources in terms of perspectives across stakeholders and across conditions (HIV/hypertension/DM/multi/co-morbidity) when raising the abstraction level. Triangulation of socio-behavioural qualitative and observational data during analysis will occur in order to understand how different types of evidence enhance the overall interpretation of how INTE-AFRICA was implemented, and what the additional health economic and clinical data are, drawing case comparisons across clinics, and across countries, and developing possible explanations for implementation variation. The data, when combined and triangulated across these multi stakeholder perspectives, will provide a *'thick description*', of how the intervention was delivered, maintained and experienced by stakeholders.[10 45] It will also offer explanations for observed variation over time and between countries, and detailed insight into the interaction between different contextual features and components of integration of NCD or HIV/NCD

For peer review only - http://bmjopen.bmj.com/site/about/guidelines.xhtml

services. It will also facilitate triangulation of information across stakeholders, clinics and countries. We envisage returning to these two clinics in the future, four years and six years after integration, to achieve a deeper understanding of processes and patient and provider experiences. This approach will help to support transferability to other settings, by identifying factors which are plausibly and/or consistently related to successful or unsuccessful delivery of intervention components.

Emerging theories and the relationship of the data to the conceptual literature underpinning the intervention will be discussed and refined at INTE-AFRICA research team meetings throughout the project. We envisage utilising the public understanding of science theory [54] to unpack how patients and their communities in Uganda and Tanzania understand and use different knowledge on HIV and NCDs in their lives. This could be facilitated by understanding how they create meaning from scientific findings relating to NCDs and HIV and if, how, and to what degree they incorporate these findings into their everyday lives. This theory has the capacity to shift public attitudes by connecting and communicating the development of innovative scientific concepts in the medical field (in this instance integration of HIV/NCD services in Tanzania and Uganda) to the non-scientific public, and thereby enhance education, training cascade, health policy and practice, and ultimately public understanding of multi-morbidities and sustainable routes to care. Further, it will create a platform for the sharing of lessons learnt, best practices and context adaptation of the final integrated model of care in other African countries (clinical care policies and practice, staff cascade of training, service user education and community awareness raising).

Ethical considerations

Ethical approval for the evaluation has been granted by the research ethics committees of the Liverpool School of Tropical Medicine (UK), the National Institute of Medical Research (Tanzania) and TASO Research Ethics Committee (Uganda). The key ethical principles of voluntary and informed participation, confidentiality and safety of participants will be used in all researcher and participant interactions. Written consent for interviews and observations will be obtained from all participants. All participants will be provided with written information about the research, this will be explained verbally, and informed that their participation is voluntary and that they may withdraw from participation at any time. Safety and confidentiality of all data will be ensured by: (1) encrypting all transcriptions with a password protected code; (2) storing all data in a secure, encrypted database accessible only to authorised persons on the research team; (3) de-linking all personal information of participants from the data collected and stored. Each participant will have a unique identification number.

For peer review only - http://bmjopen.bmj.com/site/about/guidelines.xhtml

Contributorship Statement

All authors contributed to the conceptualisation of the research and contributed to writing the manuscript.

MCVH, MB, JVL, SJ, ES, DB, CP, JO designed the process evaluation protocol.

SJ, MN, SM, JB led the development of the INTE-AFRICA trial.

MCVH drafted the manuscript and all co-authors edited and commented on subsequent drafts. All authors approved the final draft for submission. All authors agree to be accountable for all aspects of the work in ensuring that questions related to the accuracy or integrity of any part of the work are appropriately investigated and resolved.

Funding Statement

The INTE-AFRICA project has received funding from the European Union's Horizon 2020 research and innovation programme under grant agreement No 825698. JVL is supported by a Spanish Ministry of Science, Innovation and Universities Miguel Servet grant (Instituto de Salud Carlos III/ESF, European Union [CP18/00074]) and further acknowledges support from the Spanish Ministry of Science, Innovation and Universities through the "Centro de Excelencia Severo Ochoa 2019-2023" Programme (CEX2018-000806-S), and from the Government of Catalonia through the CERCA Programme.

Ethical approval statement

Ethical approval for the evaluation has been granted in 2019 at the Liverpool School of Tropical Medicine (UK), the National Institute of Medical Research (Tanzania) and TASO Research Ethics Committee (Uganda).

Data sharing statement

No additional data available.

Competing interests statement

None

Provenance and peer review

Not commissioned; internally peer reviewed.

For peer review only

Open access

This is an open access article distributed in accordance with the Creative Commons Attribution 4.0 Unported (CC BY 4.0) license, which permits others to copy, redistribute, remix, transform and build upon this work for any purpose, provided the original work is properly cited, a link to the licence is given, and indication of whether changes were made. See: https:// creative commons. org/ licenses/ by/ 4. 0/.

For peer review only

References

1. Adeloye D, Basquill C. Estimating the prevalence and awareness rates of hypertension in Africa: a systematic analysis. *PLoS One* 2014;9(8):e104300.
2. Atun R, Davies JI, Gale EAM, et al. Diabetes in sub-Saharan Africa: from clinical care to health policy. *Lancet Diabetes Endocrinol* 2017;5(8):622-67.
3. Remais JV, Zeng G, Li G, et al. Convergence of non-communicable and infectious diseases in low- and middle-income countries. *Int J Epidemiol* 2013;42(1):221-7.
4. Ford N, Ball A, Baggaley R, et al. The WHO public health approach to HIV treatment and care: looking back and looking ahead. *Lancet Infect Dis* 2018;18(3):e76-e86.
5. Global, regional, and national comparative risk assessment of 79 behavioural, environmental and occupational, and metabolic risks or clusters of risks, 1990-2015: a systematic analysis for the Global Burden of Disease Study 2015. *Lancet* 2016;388(10053):1659-724.
6. Guwatudde D, Nankya-Mutyoba J, Kalyesubula R, et al. The burden of hypertension in sub-Saharan Africa: a four-country cross sectional study. *BMC Public Health* 2015;15:1211.
7. Lim SS, Vos T, Flaxman AD, et al. A comparative risk assessment of burden of disease and injury attributable to 67 risk factors and risk factor clusters in 21 regions, 1990-2010: a systematic analysis for the Global Burden of Disease Study 2010. *Lancet* 2012;380(9859):2224-60.
8. Addo J, Smeeth L, Leon DA. Hypertension in sub-saharan Africa: a systematic review. *Hypertension* 2007;50(6):1012-8.
9. Chang AY, Gómez-Olivé FX, Manne-Goehler J, et al. Multimorbidity and care for hypertension, diabetes and HIV among older adults in rural South Africa. *Bull World Health Organ* 2019;97(1):10-23.
10. George S, McGrath N, Oni T. The association between a detectable HIV viral load and non-communicable diseases comorbidity in HIV positive adults on antiretroviral therapy in Western Cape, South Africa. *BMC Infect Dis* 2019;19(1):348.
11. Jaffar S, Gill G. The crisis of diabetes in sub-Saharan Africa. *Lancet Diabetes Endocrinol* 2017;5(8):574-75.
12. Jaffar S. Diabetes and other non-communicable diseases in Africa: a potential disaster in the waiting. *Lancet Diabetes Endocrinol* 2016;4(11):875-77.
13. Lupafya PC, Mwagomba BL, Hosig K, et al. Implementation of Policies and Strategies for Control of Noncommunicable Diseases in Malawi: Challenges and Opportunities. *Health Educ Behav* 2016;43(1 Suppl):64s-9s.
14. Katende D, Mutungi G, Baisley K, et al. Readiness of Ugandan health services for the management of outpatients with chronic diseases. *Trop Med Int Health* 2015;20(10):1385-95.
15. Manne-Goehler J, Atun R, Stokes A, et al. Diabetes diagnosis and care in sub-Saharan Africa: pooled analysis of individual data from 12 countries. *Lancet Diabetes Endocrinol* 2016;4(11):903-12.
16. Peck R, Mghamba J, Vanobberghen F, et al. Preparedness of Tanzanian health facilities for outpatient primary care of hypertension and diabetes: a cross-sectional survey. *Lancet Glob Health* 2014;2(5):e285-92.
17. Price AJ, Crampin AC, Amberbir A, et al. Prevalence of obesity, hypertension, and diabetes, and cascade of care in sub-Saharan Africa: a cross-sectional, population-based study in rural and urban Malawi. *Lancet Diabetes Endocrinol* 2018;6(3):208-22.
18. Patel P, Rose CE, Collins PY, et al. Noncommunicable diseases among HIV-infected persons in low-income and middle-income countries: a systematic review and meta-analysis. *Aids* 2018;32 Suppl 1(Suppl 1):S5-s20.
19. Haregu TN, Setswe G, Elliott J, et al. Integration of HIV/AIDS and noncommunicable diseases in developing countries: rationale, policies and models. *International Journal of Healthcare* 2015;1:21-27.

For peer review only - http://bmjopen.bmj.com/site/about/guidelines.xhtml

20. Husain NE, Noor SK, Elmadhoun WM, et al. Diabetes, metabolic syndrome and dyslipidemia in people living with HIV in Africa: re-emerging challenges not to be forgotten. *HIV AIDS (Auckl)* 2017;9:193-202.
21. Juma K, Reid M, Roy M, et al. From HIV prevention to non-communicable disease health promotion efforts in sub-Saharan Africa: A Narrative Review. *Aids* 2018;32 Suppl 1:S63-s73.
22. Haldane V, Legido-Quigley H, Chuah FLH, et al. Integrating cardiovascular diseases, hypertension, and diabetes with HIV services: a systematic review. *AIDS Care* 2018;30(1):103-15.
23. Rabkin M, Melaku Z, Bruce K, et al. Strengthening Health Systems for Chronic Care: Leveraging HIV Programs to Support Diabetes Services in Ethiopia and Swaziland. *J Trop Med* 2012;2012:137460.
24. Some D, Edwards JK, Reid T, et al. Task Shifting the Management of Non-Communicable Diseases to Nurses in Kibera, Kenya: Does It Work? *PLoS One* 2016;11(1):e0145634.
25. Craig P, Dieppe P, Macintyre S, et al. Developing and evaluating complex interventions: the new Medical Research Council guidance. *Bmj* 2008;337:a1655.
26. Iwelunmor J, Ezechi O, Obiezu-Umeh C, et al. Capabilities, opportunities and motivations for integrating evidence-based strategy for hypertension control into HIV clinics in Southwest Nigeria. *PLoS One* 2019;14(6):e0217703.
27. Rachlis B, Naanyu V, Wachira J, et al. Identifying common barriers and facilitators to linkage and retention in chronic disease care in western Kenya. *BMC Public Health* 2016;16:741.
28. Ameh S, Klipstein-Grobusch K, Musenge E, et al. Effectiveness of an Integrated Approach to HIV and Hypertension Care in Rural South Africa: Controlled Interrupted Time-Series Analysis. *J Acquir Immune Defic Syndr* 2017;75(4):472-79.
29. Anand TN, Joseph LM, Geetha AV, et al. Task sharing with non-physician health-care workers for management of blood pressure in low-income and middle-income countries: a systematic review and meta-analysis. *Lancet Glob Health* 2019;7(6):e761-e71.
30. Divala OH, Amberbir A, Ismail Z, et al. The burden of hypertension, diabetes mellitus, and cardiovascular risk factors among adult Malawians in HIV care: consequences for integrated services. *BMC Public Health* 2016;16(1):1243.
31. Edwards JK, Bygrave H, Van den Bergh R, et al. HIV with non-communicable diseases in primary care in Kibera, Nairobi, Kenya: characteristics and outcomes 2010-2013. *Trans R Soc Trop Med Hyg* 2015;109(7):440-6.
32. Golovaty I, Sharma M, Van Heerden A, et al. Cost of Integrating Noncommunicable Disease Screening Into Home-Based HIV Testing and Counseling in South Africa. *J Acquir Immune Defic Syndr* 2018;78(5):522-26.
33. Kwarisiima D, Atukunda M, Owaraganise A, et al. Hypertension control in integrated HIV and chronic disease clinics in Uganda in the SEARCH study. *BMC Public Health* 2019;19(1):511.
34. Fairall LR, Folb N, Timmerman V, et al. Educational Outreach with an Integrated Clinical Tool for Nurse-Led Non-communicable Chronic Disease Management in Primary Care in South Africa: A Pragmatic Cluster Randomised Controlled Trial. *PLoS Med* 2016;13(11):e1002178.
35. Mahomed OH, Asmall S. Development and implementation of an integrated chronic disease model in South Africa: lessons in the management of change through improving the quality of clinical practice. *Int J Integr Care* 2015;15:e038.
36. Matanje Mwagomba BL, Ameh S, Bongomin P, et al. Opportunities and challenges for evidence-informed HIV-noncommunicable disease integrated care policies and programs: lessons from Malawi, South Africa, Swaziland and Kenya. *Aids* 2018;32 Suppl 1:S21-s32.
37. Nuche-Berenguer B, Kupfer LE. Readiness of Sub-Saharan Africa Healthcare Systems for the New Pandemic, Diabetes: A Systematic Review. *J Diabetes Res* 2018;2018:9262395.
38. Pfaff C, Scott V, Hoffman R, et al. You can treat my HIV - But can you treat my blood pressure? Availability of integrated HIV and non-communicable disease care in northern Malawi. *Afr J Prim Health Care Fam Med* 2017;9(1):e1-e8.

For peer review only

For peer review only - http://bmjopen.bmj.com/site/about/guidelines.xhtml

39. Pfaff C, Singano V, Akello H, et al. Early experiences integrating hypertension and diabetes screening and treatment in a human immunodeficiency virus clinic in Malawi. *Int Health* 2018;10(6):495-501.
40. Rawat A, Uebel K, Moore D, et al. Integrated HIV-Care Into Primary Health Care Clinics and the Influence on Diabetes and Hypertension Care: An Interrupted Time Series Analysis in Free State, South Africa Over 4 Years. *J Acquir Immune Defic Syndr* 2018;77(5):476-83.
41. Jaffar S, Amuron B, Foster S, et al. Rates of virological failure in patients treated in a home-based versus a facility-based HIV-care model in Jinja, southeast Uganda: a cluster-randomised equivalence trial. *Lancet* 2009;374(9707):2080-89.
42. Georgeu D, Colvin CJ, Lewin S, et al. Implementing nurse-initiated and managed antiretroviral treatment (NIMART) in South Africa: a qualitative process evaluation of the STRETCH trial. *Implement Sci* 2012;7:66.
43. Moore G, Audrey S, Barker M, et al. Process evaluation in complex public health intervention studies: the need for guidance. *J Epidemiol Community Health* 2014;68(2):101-2.
44. Linnan L, Steckler A. Process evaluation for public health interventions and research. An overview. In: Steckler A, Linnan L, eds. Process evaluation for public health interventions and research. 1st ed. San Francisco: Josset-Bass 2002:1-23.
45. Murdoch J, Curran R, Bachmann M, et al. Strengthening the quality of paediatric primary care: protocol for the process evaluation of a health systems intervention in South Africa. *BMJ Glob Health* 2018;3(Suppl 5):e000945.
46. Kippax S. Effective HIV prevention: the indispensable role of social science. *J Int AIDS Soc* 2012;15(2):17357.
47. Bronfenbrenner U. Toward an experimental ecology of human development. *American Psychologist* 1977;32(7):513-31.
48. Bronfenbrenner U. The ecology of human development: Experiments by nature and design. Cambridge, MA: Harvard University Press 1979.
49. Hawe P, Shiell A, Riley T. Theorising interventions as events in systems. *Am J Community Psychol* 2009;43(3-4):267-76.
50. Grimshaw JM, Zwarenstein M, Tetroe JM, et al. Looking inside the black box: a theory-based process evaluation alongside a randomised controlled trial of printed educational materials (the Ontario printed educational message, OPEM) to improve referral and prescribing practices in primary care in Ontario, Canada. *Implement Sci* 2007;2:38.
51. Karlsson G. Psychological Qualitative Research From a Phenomenological Perspective: Almqvist & Wiksell International 1993.
52. Marshall C, Rossman GB. Designing qualitative research. 4th ed. Thousands Oaks, Calif: Sage Publications 2006.
53. Patton MQ, Patton MQ. Qualitative research and evaluation methods. Thousand Oaks, Calif.: Sage Publications 2002.
54. Brossard D, Lewenstein B. A Critical Appraisal of Models of Public Understanding of Science: Using Practice to Inform Theory2010:11-39.

For peer review only

For peer review only - http://bmjopen.bmj.com/site/about/guidelines.xhtml

Table One 'Key data on the country settings'

	Tanzania	Uganda
Income level	Low	Low
Population size	58m (2018)	35m (2016)
Estimated prevalence of hypertension from STEPS survey	26%	26%
Estimated prevalence of diabetes from STEPS survey [15] *	5-10%	2-5%
Estimated prevalence of HIV-infection	5.1% (2017)	6.2% (2017)
Doctors density /100,000 population	3 (2014)	0.8 (2005)

* diabetes estimate varies according to age and gender. Data are of variable quality but reference 11 shows that the overall median diabetes prevalence in 12 countries in Africa is 5%.

For peer review only

For peer review only - http://bmjopen.bmj.com/site/about/guidelines.xhtml

Table Two 'Logic Model of programme inputs, processes and outcomes'

<table>
<tr><th>Intervention inputs</th><th>Changes in care processes</th><th>Outcomes</th></tr>
<tr><td>Negotiation with national, district and local government health departments, NGOs and funders</td><td>Agreement about and support for service model, including reorganisation of clinics and staff
Commitment to ensure drug supply</td><td>• Reorganisation of clinics and staff to implement the model
• An effective, quality, and sustainable funded drug supply chain</td></tr>
<tr><td>Negotiating lower drug prices
Supporting and monitoring drug ordering in each clinic
Providing buffer drug supplies to each clinic</td><td>Drugs always in stock</td><td rowspan="6">• Increased diagnosis of comorbid conditions
• Increased retention and adherence
• Increased viral suppression, better control of blood pressure and blood glucose
• Less AIDS, cardiovascular disease and diabetes complications
• Lower patients costs (travel and absence from work)
• Less health service duplication and costs (health service costs might increase if more patients are diagnosed and are more adherent)
• Increase patient satisfaction
• Increased clinician satisfaction; less burn-out and absenteeism
• Reduce missed opportunities for improving care and health outcomes</td></tr>
<tr><td>Engagement with and support for clinicians and managers in each clinic</td><td>Clinicians and managers enable and support integration, and find solutions to emerging problems</td></tr>
<tr><td>Provision of integrated service in each clinic (alongside and additional to existing services)</td><td>Trial participants attend integrated service
Avoid multiple visits for patients with multi-morbidity</td></tr>
<tr><td>Training clinicians about integrated clinical management</td><td>Better diagnosis and treatment including attention to comorbid conditions</td></tr>
<tr><td>Community engagement</td><td>Identify and enlist community organisations and resources to help with health education, tracing defaulters or patients who have difficulty attending clinic</td></tr>
<tr><td>Providing standardised stationery for integrated medical records; training clinicians to use it</td><td>Increased awareness by clinicians and patients about disease severity, comorbidity, adherence and control in individual patients</td></tr>
<tr><td>Improving monitoring and evaluation based on clinics registers and medical records</td><td>Regular data analysis and feedback to staff</td><td>Quality assurance and continuous improvement in the quality of care</td></tr>
<tr><td>Identifying effective health education</td><td>Improve health education at clinics</td><td>Healthier lifestyles
Increased adherence</td></tr>
</table>

For peer review only - http://bmjopen.bmj.com/site/about/guidelines.xhtml

Table Three: 'Process Evaluation Design and data collection framework'

**Post Integration**<br>*** Numbers indicated are per country/.**<br>**Data Collection at each Site**	**6 months**	**12 months**	**18 months**	**Contextual level**
Observations of consultations, different processes and clinic flow at clinic levels and in non-clinical areas.	1 week	1 week	1 week	**Micro context (facility and neighbourhoods)**
In depth phenomenological interviews with patients/service users	25	25	25	**Micro context (facility and neighbourhoods)**
In depth phenomenological interviews with health care providers at the clinic (hospital overall in charge, hospital pharmacist, the medical officers in-charge of the integrated clinic, trained clinicians managing HIV, diabetes and hypertension patients, pharmacist, laboratory technician, counsellors or nurses providing health education and counselling and nurses in the registration desk who are also responsible in taking vital signs)	10	10	10	**Meso context (national to regional/city)**<br>**Micro context (facility and neighbourhoods)**
Semi-structured interviews with Ministerial policy makers and provincial/regional/district level clinical/health senior management (Director for NCD, HIV and curative services).	-	5	5	**Macro context (Global)**<br>**Meso context (national to regional/city)**
Semi-structured interviews with NGO and international organisations (for example WHO Country office, UNAIDS, PEPFAR, CDC)	-	5	5	**Macro context (Global)**<br>**Meso context (national to regional/city)**
Focus group discussions (FGD) with community leaders (8-12 participants)	1	1	1	**Micro context (facility and neighbourhoods)**
Focus group discussions (FGD) gender specific with community members(8-12participants)	2	2	2	**Micro context (facility and neighbourhoods)**
In depth phenomenological interviews with clinical researchers			4	**Micro context (facility and neighbourhoods)**<br>**Meso context (national to regional/city)**

For peer review only - http://bmjopen.bmj.com/site/about/guidelines.xhtml

Figure One ‘Potential benefits of integrating diabetes, hypertension and HIV services for a) DM and hypertension control, and b) HIV control’

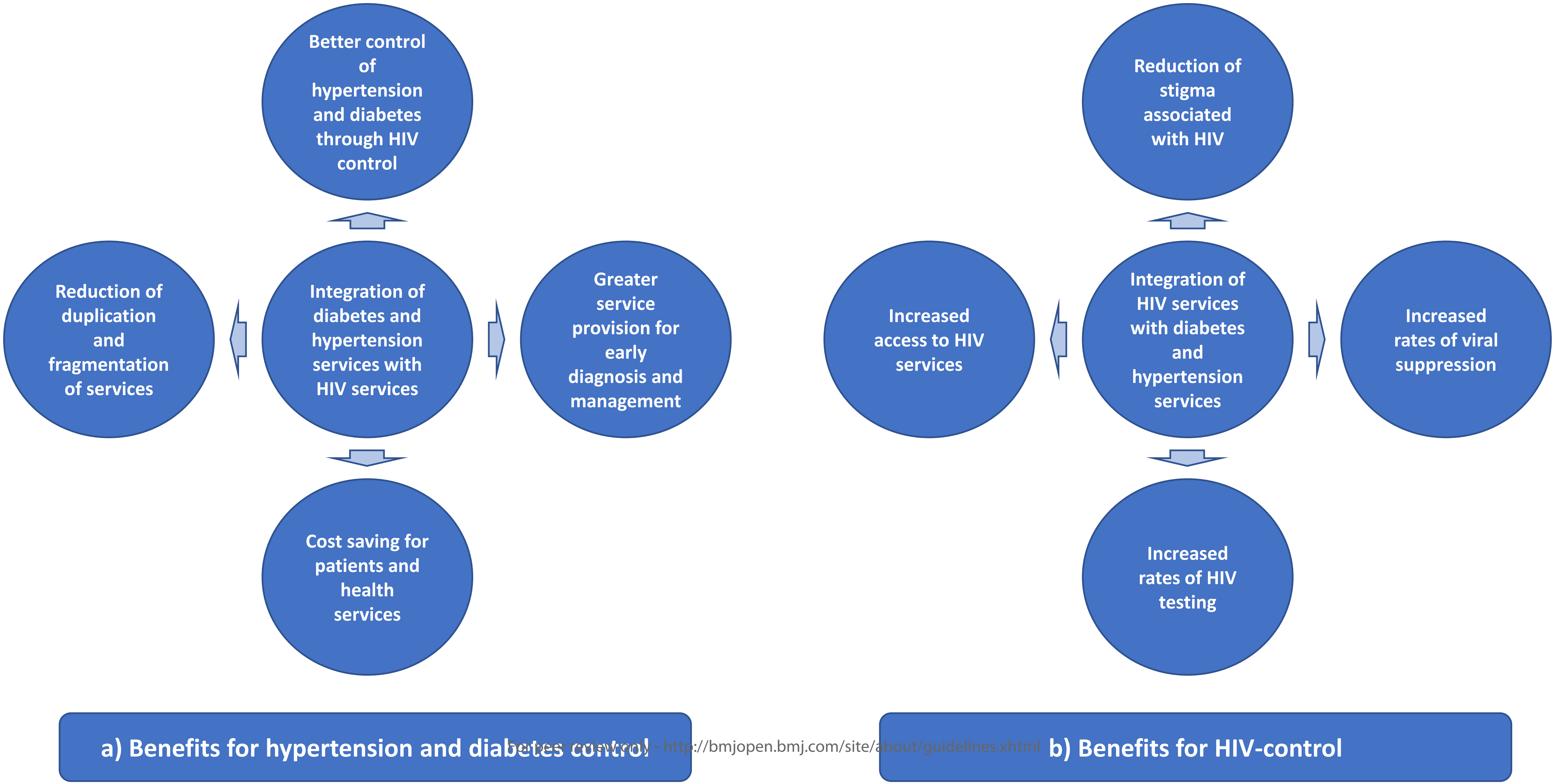

For peer review only - http://bmjopen.bmj.com/site/about/guidelines.xhtml

Figure Two 'INTEAFRICA Conceptual Model'

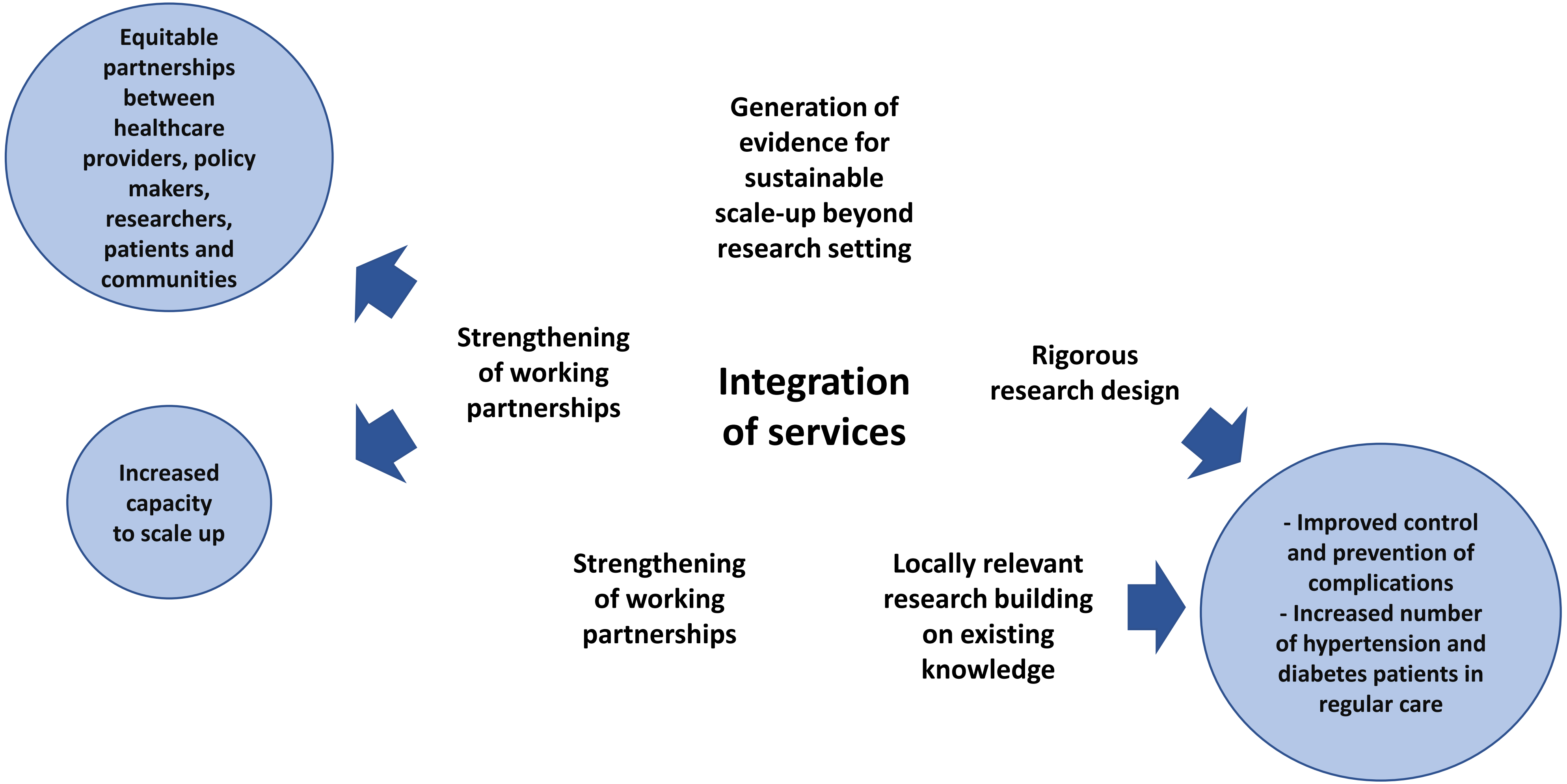

Figure Three ‘Potential contextual influences on INTEAFRICA programme implementation cascade’

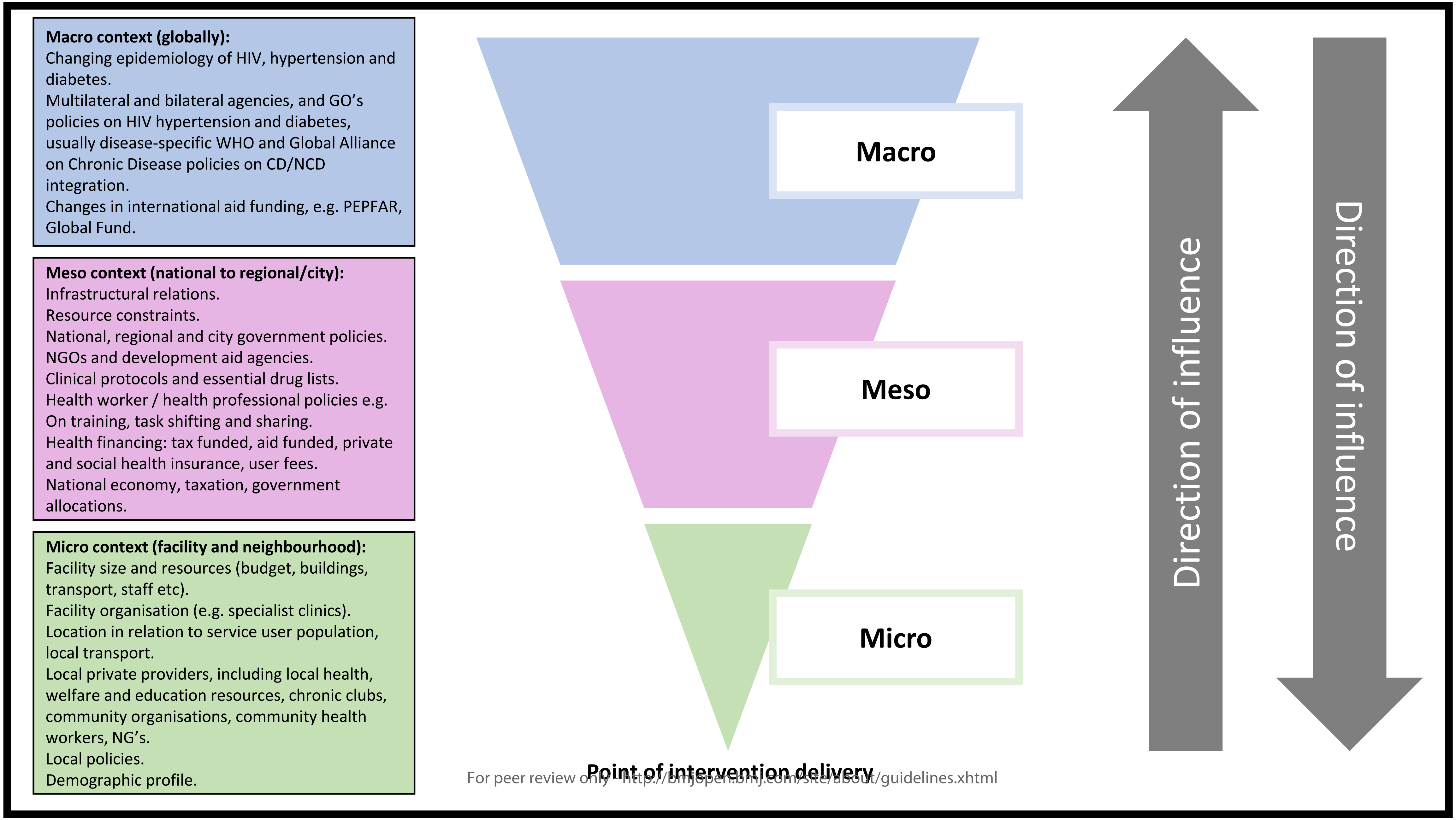

For peer review only - http://bmjopen.bmj.com/site/about/guidelines.xhtml

Strengthening integration of chronic care in Africa: protocol for the qualitative process evaluation of integrated HIV, diabetes and hypertension care in a cluster randomised controlled trial in Tanzania and Uganda.

Journal:	*BMJ Open*
Manuscript ID	bmjopen-2020-039237.R2
Article Type:	Protocol
Date Submitted by the Author:	26-Aug-2020

Complete List of Authors:	Van Hout, Marie-Claire; Liverpool John Moores University, Public Health Institute<br>Bachmann, Max; University of East Anglia Faculty of Medicine and Health Sciences, Norwich Medical School<br>Lazarus, Jeffrey; Instituto de Salud Global Barcelona, Hospital Cliínic, University of Barcelona;<br>Shayo, Elizabeth; National Institute for Medical Research, Muhimbili Centre<br>Bukenya, Dominic; Medical Research Council Uganda, MRC/UVRI/LSHTM Uganda Research Unit<br>Picchio, Camila; Instituto de Salud Global Barcelona, Hospital Clinic University of Barcelona<br>Nyirenda, Moffat; Medical Research Council Uganda , MRC/UVRI/LSHTM Uganda Research Unit<br>Mfinanga, Sayoki; National Institute for Medical Research, Muhimbili Centre<br>Birungi, Josephine; Medical Research Council Uganda , MRC/UVRI/LSHTM Uganda Research Unit<br>Okebe, Joseph; Liverpool School of Tropical Medicine, Department of International Public Health<br>Jaffar, Shabbar; Liverpool School of Tropical Medicine, Department of International Public Health

**Primary Subject Heading**:	HIV/AIDS
Secondary Subject Heading:	Global health, Health services research, Patient-centred medicine, Diabetes and endocrinology
Keywords:	Hypertension < CARDIOLOGY, DIABETES & ENDOCRINOLOGY, International health services < HEALTH SERVICES ADMINISTRATION & MANAGEMENT, Organisation of health services < HEALTH SERVICES ADMINISTRATION & MANAGEMENT

For peer review only - http://bmjopen.bmj.com/site/about/guidelines.xhtml

SCHOLARONE™
Manuscripts

I, the Submitting Author has the right to grant and does grant on behalf of all authors of the Work (as defined in the below author licence), an exclusive licence and/or a non-exclusive licence for contributions from authors who are: i) UK Crown employees; ii) where BMJ has agreed a CC-BY licence shall apply, and/or iii) in accordance with the terms applicable for US Federal Government officers or employees acting as part of their official duties; on a worldwide, perpetual, irrevocable, royalty-free basis to BMJ Publishing Group Ltd ("BMJ") its licensees and where the relevant Journal is co-owned by BMJ to the co-owners of the Journal, to publish the Work in this journal and any other BMJ products and to exploit all rights, as set out in our licence.

The Submitting Author accepts and understands that any supply made under these terms is made by BMJ to the Submitting Author unless you are acting as an employee on behalf of your employer or a postgraduate student of an affiliated institution which is paying any applicable article publishing charge ("APC") for Open Access articles. Where the Submitting Author wishes to make the Work available on an Open Access basis (and intends to pay the relevant APC), the terms of reuse of such Open Access shall be governed by a Creative Commons licence – details of these licences and which Creative Commons licence will apply to this Work are set out in our licence referred to above.

Other than as permitted in any relevant BMJ Author's Self Archiving Policies, I confirm this Work has not been accepted for publication elsewhere, is not being considered for publication elsewhere and does not duplicate material already published. I confirm all authors consent to publication of this Work and authorise the granting of this licence.

For peer review only

Title

Strengthening integration of chronic care in Africa: protocol for the qualitative process evaluation of integrated HIV, diabetes and hypertension care in a cluster randomised controlled trial in Tanzania and Uganda.

Author Listing

Marie Claire Van Hout, Liverpool John Moores University, Public Health Institute, United Kingdom

Max Bachmann, University of East Anglia, Faculty of Medicine and Health Sciences, United Kingdom

Jeffrey V Lazarus, Instituto de Salud Global Barcelona, Barcelona Institute for Global Health (ISGlobal), Hospital Clínic, University of Barcelona, Spain

Elizabeth H. Shayo, National Institute for Medical Research (NIMR), Muhimbili Centre, Tanzania

Dominic Bukenya, Medical Research Council, MRC/UVRI/LSHTM Uganda Research Unit, Uganda

Camila A Picchio, Instituto de Salud Global Barcelona (Barcelona Institute for Global Health ISGlobal), Hospital Clínic, University of Barcelona, Spain

Moffat Nyirenda, Medical Research Council, MRC/UVRI/LSHTM Uganda Research Unit, Uganda

Sayoki Mfinanga, National Institute for Medical Research (NIMR), Muhimbili Centre, Tanzania; and Liverpool School of Tropical Medicine, Department of International Public Health, United Kingdom, Muhimbili University of Health and Allied Sciences, Department of Epidemiology and Biostatistics.

Josephine Birungi, Medical Research Council, MRC/UVRI/LSHTM Uganda Research Unit, Uganda

Joseph Okebe, Liverpool School of Tropical Medicine, Department of International Public Health, United Kingdom

Shabbar Jaffar, Liverpool School of Tropical Medicine, Department of International Public Health, United Kingdom

Corresponding Author:

Professor Marie Claire Van Hout, Public Health Institute, Exchange Station, Liverpool John Moores University, Liverpool L32ET, United Kingdom. Email: m.c.vanhout@ljmu.ac.uk

Article Type: Protocol

For peer review only

Abstract

Introduction In sub-Saharan Africa (SSA), the burden of non-communicable diseases (NCDs), particularly diabetes mellitus (DM) and hypertension, has increased rapidly in recent years, although HIV infection remains a leading cause of death among young-middle-aged adults. Health service coverage for NCDs remains very low in contrast to HIV, despite the increasing prevalence of comorbidity of NCDs with HIV. There is an urgent need to expand healthcare capacity to provide integrated services to address these chronic conditions.

Methods and Analysis: This protocol describes procedures for a qualitative process evaluation of INTE-AFRICA, a cluster-randomised trial comparing integrated health service provision for HIV-infection, DM, and hypertension, to the current stand-alone vertical care. Interviews, focus group discussions, and observations of consultations and other care processes in two clinics (in Tanzania, Uganda) will be used to explore the experiences of stakeholders. These stakeholders will include health service users, policy-makers, healthcare providers, community leaders and members, researchers, non-governmental (NGO) and international organisations. The exploration will be carried out during the implementation of the project, alongside an understanding of the impact of broader structural and contextual factors.

Ethics and Dissemination: Ethical approval was granted by the Liverpool School of Tropical Medicine (UK), the National Institute of Medical Research (Tanzania) and TASO Research Ethics Committee (Uganda) in 2020. The evaluation will provide the opportunity to document the implementation of integration over several timepoints (6, 12 and 18 months) and refine integrated service provision prior to scale-up. This synergistic approach to evaluate, understand and respond will support service integration and inform monitoring, policy and practice development efforts to involve and educate communities in Tanzania and Uganda. It will create a model of care and a platform of good practices and lessons learnt for other countries implementing integrated and decentralised community health services.

Key Words

Health service delivery, Non communicable disease, HIV, integration, task-shifting, sub-Saharan Africa

For peer review only - http://bmjopen.bmj.com/site/about/guidelines.xhtml

Article Summary: Strengths and Limitations of this study

- The INTE-AFRICA trial will implement integration of HIV/NCD services in Tanzania and Uganda in response to an urgent need to respond to increased burden of NCDs, and expand capacity of healthcare systems to manage comorbidity.
- The INTE-AFRICA trial is based on a partnership between African and European researchers, working closely with policy-makers and other stakeholders.
- The process evaluation of INTE-AFRICA employs qualitative and observational methods at two facilities to explore and document stakeholder experiences of integration of services, alongside an understanding of the impact of broader structural and contextual factors.
- The process evaluation works in tandem with quantitative evaluation of clinical efficacy and cost-effectiveness of the INTE-AFRICA trial.
- Limitations of the process evaluation may centre on patient drop-out, characteristics of the two selected sites, selection, information and social desirability bias and other external mitigating factors.

For peer review only

BACKGROUND

Non-communicable diseases (NCDs) have risen rapidly in Africa, alongside a continuing high burden of HIV-infection. In sub-Saharan Africa (SSA), HIV/AIDS remains a major cause of morbidity and mortality among young-middle aged adults, but the region is also experiencing a rapidly rising burden of NCDs (particularly DM and hypertension), giving rise to a dual HIV-NCD epidemic.[1-3] While lifestyle changes associated with urbanisation and globalization (such as eating habits and lack of physical exercise) underpin the ongoing demographic and epidemiologic transition toward increasing NCDs, these changes also affect chronic conditions (such as HIV).[1-3] The number of people in regular HIV care is rising[4], alongside the rate of NCDs in the SSA region, with hypertension representing the single largest risk factor for death, and DM which has seen a massive increase in prevalence in a short period of time.[2 5-7] Patient populations in SSA are increasingly demonstrating younger age of onset of NCDs, with comorbidity of NCDs with HIV, and the impact of NCDs is particularly severe in populations affected by poverty.[3 8-11]

Since 2003, significant global investment and development partner engagement has facilitated the establishment of HIV screening and treatment programmes as the first large-scale chronic disease initiatives in Africa. Health services for HIV are stand-alone and vertically delivered. They have also been combined with decentralisation and task-shifting, which has enabled primary health centres to treat large numbers of patients. Non-clinically qualified health workers play a major role in supporting patients on HIV treatment, with almost 70% of people living with HIV-infection in regular care. In contrast, health service coverage for NCDs remains very low.[2 12] Coordinated national NCD control and care programmes are relatively new with significant gaps in funding and operational evidence for program implementation.[11 13.] For example, only about 5-10% of persons with DM are thought to be in regular diabetes care, and the figure is likely to be similar for persons with hypertension.[2 8 11] Furthermore, those in care experience insufficient health service provision for the diagnosis and management of DM and hypertension (medicines supply is patchy).[2 12 14-17]. HIV populations at risk of developing NCDs, and presenting with co/multi-morbidities could potentially impact on the gains from the achieved scale-up of HIV care services.[18]

Hence, there is an urgent need to expand the capacity of healthcare systems in the SSA region to provide services for NCDs, either alongside, or integrated, with HIV.[19] As chronic conditions, DM, hypertension and HIV-infection require lifelong care. Key challenges to chronic care in SSA are linkage and retention in care, access and medicines adherence.[20 21] As such, the infrastructure and lessons learnt from the HIV chronic disease model can serve as important resources for the expansion of NCD prevention, care and treatment. HIV chronic care management pathways, resources and infrastructure can be leveraged to integrate with newly developing NCD services, ultimately to strengthen the platform for NCD services and improve health outcomes for people with NCDs.[22-24] Health systems have the developed experience

For peer review only - http://bmjopen.bmj.com/site/about/guidelines.xhtml

managing HIV as a chronic disease, including linking and retaining patients in care and supporting treatment adherence in drugs, diagnostics procurement and other key health systems indicators.[21 25-27] Integration can reduce duplication and fragmentation, streamline services by treat those with co/multi-morbidities, and potentially offer patient benefits relating to time and cost. See **Figure 1**. It could however also threaten service capacity by resulting in increased service demand and loss of clinical focus on one disease.

Figure 1 'Potential benefits of integrating diabetes, hypertension and HIV services for a) DM and hypertension control, and b HIV control'

Despite the increase in academic and clinical interest in HIV/NCD integration in SSA, [9 26 28-33] little evidence about integration in terms of both scope and generalizability exists. Extant evidence is limited to small scale feasibility studies in largely different contexts [34-40], despite suggesting that integrated care is an efficient use of resources compared to the standard of care and beneficial for patients' NCD and HIV clinical outcomes.[28 30 32] There is a lack of large scale or randomized and/or controlled evaluations and context-specific clinical, cost effectiveness and process outcomes data constrains policymaking and development of integrated care models, which could strengthen health systems when tailored to the distinct needs of each specific SSA country.[19 21 37] Such evidence is paramount to inform policy, government resource prioritisation, and to develop integrated care models which strengthen health systems best suited to the needs of that specific SSA country. The INTE-AFRICA trial is conducted to respond to the insufficient existing evidence to substantiate the benefits and sustainability of integrated care as well as the implementation of such programmes in the SSA setting. We present here the qualitative process evaluation protocol designed to evaluate INTE-AFRICA, a European Commission Horizon 2020 funded implementation research project (protocol number 19-100) operating in Tanzania and Uganda.

The INTE-AFRICA Trial

INTE-AFRICA aims to implement and assess the effectiveness of the integration of HIV, DM and hypertension services at the point of service delivery covering many health facilities where common approaches to clinical decision-making, drug procurement and human resource management occur. The project will generate the research evidence needed by health services in Africa, to scale-up and sustain chronic disease management and services in an integrated manner. We are also interested in observing changes in dynamics pertaining to stigma and discrimination associated with HIV.[41]

INTE-AFRICA team specifically chose Tanzania and Uganda as they are low-income countries and their public and private health facilities are strongly committed to providing services for NCD. However, their health systems struggle to scale-up provision for diabetes and hypertension in the face

For peer review only - http://bmjopen.bmj.com/site/about/guidelines.xhtml

of competing health demands, including HIV-infection. See **Table 1**. Tanzania and Uganda also share relevant characteristics with other countries in SSA. As such, the process evaluation has the potential to enhance the generalisability of integrated care to other similar settings by providing understanding of the determinants and mechanisms of the implementation process.

Table 1 'Key data on the country settings'

Our programme is underpinned by a participatory, multi-actor approach which supports dialogue and knowledge exchange, fosters mutual understanding and provides input in policy agendas around diagnosis and treatment of DM, hypertension and HIV in an integrated clinic. The INTE-AFRICA conceptual framework is illustrated in **Figure 2.**

Figure 2 'INTEAFRICA Conceptual Model'

INTE-AFRICA will test integrated health services at primary care centres for HIV-infection, diabetes and hypertension, by providing a "*one-stop*" integrated care clinic for these conditions (the intervention). We will conduct a pragmatic parallel arm cluster-randomised trial: 32 largely urban health facilities offering primary care services in the two countries will be randomised, with 16 facilities allocated to deliver the intervention immediately (intervention arm), and 16 facilities to continue with usual care (control arm). At each selected facility, cohorts of approximately 220 patients; 110 HIV infected and 110 NCD patients, will be enrolled to evaluate the primary research outcomes.

The primary care facilities will initiate and stabilise patients on treatment, manage complications (including referral to higher facilities) and conduct clinical and laboratory monitoring of patients for all three conditions. Specific characteristics of integrated care in each '*one stop'* integrated care clinic are: concurrent management of HIV, hypertension and DM in the same facility; management of patients with HIV, hypertension and DM by the same clinician or team of clinicians (nurses, counsellors other staff); integrated training of clinicians; single waiting area and queue; integrated health education about all three conditions; one pharmacy with a single drug dispensing point; similar testing, and cross-testing, for diagnosis and monitoring with the requisition of laboratory tests in the same place; and similar format of paper medical records for each condition kept in the same patient folder.

Patients who decline to participate in the research and trial participants in control arm clinics will continue to receive standard vertical health care delivery. Inclusion criteria for participation are: being over 18 years old; having confirmed HIV-infection, DM or hypertension; living within the catchment population of the health facility; likely to remain in the catchment population for six months and willing to provide written informed consent. Very ill patients requiring in-patient care will be excluded. The

For peer review only - http://bmjopen.bmj.com/site/about/guidelines.xhtml

research team will observe participants during clinic visits with additional reviews at six and 12 months. Further details are provided in the full INTE-AFRICA trial protocol.

Qualitative Process Evaluation of the INTE AFRICA trial

The aim of the process evaluation of INTE-AFRICA is to explore the experiences, attitudes and practices of a wide variety of stakeholders during the process of programme implementation and to develop an understanding of the impact of broader structural and contextual factors on the implementation process of service integration. Process evaluations typically evaluate how and whether interventions are delivered as intended and whether such implementation is congruent with the theory underpinning the intervention.[25 42-44] Updated Medical Research Council (MRC) guidance for evaluation of complex health interventions has recently recognised the value of process evaluation within trials stating, "*it can be used to assess fidelity and quality of implementation, clarify causal mechanisms and identify contextual factors associated with variation in outcomes.*" [25] Hence, the process evaluation in INTE-AFRICA is particularly focused on context, description of the intervention and its causal assumptions, implementation, mechanisms of impact and outcomes. [25] Further we evaluate the extent to which resources and activities supporting the intervention function to deliver intended outputs, with subsequent improvements in outcomes.

A central focus lies in identifying contextually relevant strategies for successful implementation of service integration, and practical difficulties in adoption, delivery and maintenance to inform wider implementation.[45] We recognise that outcomes (e.g. knowledge gained) of INTE-AFRICA are dependent on understanding cultures and contexts of the stakeholders (i.e. patients, healthcare providers, policy-makers, community leaders/members, non-governmental organisations (NGO), international organisations, and clinical researchers) involved and those surrounding service design and delivery as well as care seeking practices.

The role of social and behavioural science approaches to understanding individual experience, and the role of such contextual dynamics is central to this process evaluation. It aims to enhance understanding of issues related to the NCD and HIV agendas and service delivery approaches from all perspectives and stakeholders along the integration process. INTE-AFRICA will utilise a broad social behavioural approach to support community engagement in research in Tanzania and Uganda, going beyond clinical trial recruitment and retention to improve NCD/ HIV literacy. Behavioural and social science research strategies [46] will allow NCD and HIV related research to place the needs and perspectives of people living with NCD and/or HIV at the centre of the progression of clinical studies, such as INTE-AFRICA, and investigate participant motivations and decision-making processes related to preferences for or participation in different types of integrated and decentralised services.

For peer review only - http://bmjopen.bmj.com/site/about/guidelines.xhtml

Theoretical Framework: Bronfenbrenner's ecological model of behaviour

Process evaluation design, like healthcare interventions, requires a theoretical framework to structure the qualitative and observational evaluation across sites. The chosen theoretical framework is Bronfenbrenner's ecological model of behaviour [47 48] used to conceptualise integrated care as events that disrupt complex social systems[49] operating across multiple contextual levels. We are interested in better understanding the importance of context, which becomes especially relevant when comparing Tanzania and Uganda. For example, healthcare coverage and charging dynamics between countries and between HIV as opposed to NCD care differs. In Tanzania, whilst HIV services and drugs for HIV are free, patients are required to pay a direct user fee (unless elderly or very poor) or use their health insurance to pay for NCD drugs. Currently there is a strategy to progress to single health insurance to support universal health coverage in Tanzania. In Uganda, HIV services are also free to all (public and private not for profit health facilities) and HIV drugs are always in stock. However, in Uganda whilst NCD services are free in public facilities, stockouts of laboratory reagents and NCD drugs are frequent, and patients have to pay a direct user fee to access them. There is also no national insurance scheme in Uganda, only private insurance which is relatively expensive and optional.

Using the Bronfenbrenner theoretical framework, contextual factors are evaluated at the ***macro*** (universal vs partial coverage; free primary healthcare and drugs, affordability and users fees; fiscal policy, financial aspects at government level, funding barriers for chronic care management and barriers to government scale-up of service integration); ***meso*** (HIV/NCD drug ordering, drug delivery systems and continuity of supply, health care provider education and employment, community understanding and perspectives on multimorbidity); and ***micro*** (clinic and pharmacy level management, resources, patient/service user experience) levels. See **Figure 3.** These contextual factors, likely to influence implementation of integration and their effects, will be investigated to capture variation in adoption, delivery and maintenance outcomes as well as responses to the intervention. This will affect both reach and fidelity, which are hypothesised to be important factors in outcome differences.

Figure 3 'Potential contextual influences on INTE AFRICA programme implementation cascade'

To situate INTE-AFRICA within the Bronfenbrenner theoretical framework, we will develop a logic model. This model will set out contextual determinants of HIV and NCD care management in SSA and assess how integrated care components function to address these determinants to improve outcomes in the management and support of patients. See **Table 2.**

Table 2 'Logic Model of programme inputs, processes and outcomes'

For peer review only - http://bmjopen.bmj.com/site/about/guidelines.xhtml

METHODS

Study Setting

This will be a cohort study taking place in one "*one stop*" integrated care clinic per country. In Tanzania, the selected site is *Temeke Regional Referral Hospital* in Dar Es Salaam, a public tertiary health facility, with 465 staff, and serving a population of over 1 million. In Uganda, the selected site is the *Kasangati Health Centre IV*, Kasangati, a public district health facility located in the Wakiso District serving a population of over 2 million. Both provide a range of services to the community (primary care, HIV/AIDs, NCDs, surgical, maternal and child health, health education, dental and pharmacy).

Study Design

This protocol describes procedures for a qualitative process evaluation of the INTE-AFRICA pragmatic parallel arm cluster-randomised trial comparing integrated health service provision for HIV-infection, DM and hypertension with the current standard vertical care delivery model. The process evaluation works in tandem with the collection of selected clinical outcomes (e.g. clinical efficacy of different treatments) and health economic data (e.g. costs and benefits of different approaches) to estimate the potential benefits to patients and health services at clinic and country level (protocol reported elsewhere).

Patient and Public Involvement

Patient and public involvement (PPI) throughout a programme of research enhances research quality and relevance by providing different perspectives and a sense of ownership. This protocol will adhere to the same principles, and will allow the voice of '*service users*' and those affected to be heard, and utilised. Key stakeholders such as patients as service users and their families will be fully involved in guiding the research, acting as research participants, and in implementation of change in health service delivery and integrated care planning. All aspects of the process evaluation are underpinned by participatory action health research and its success and usefulness will be grounded in PPI, participation and engagement in the form of patient/professional identification of research priorities, collaborations and partnerships, expert steering, community participation around health needs and optimal integrated services, awareness raising activities, development of print materials, toolkits and training for healthcare professionals.

It will use three qualitative research techniques: in-depth interviews with stakeholders (patients, healthcare provider, policy-maker, non-governmental organisation (NGO)/international organisation, and clinical researcher); focus group discussions (FGD) with community leaders and community members; and clinic-based observations in one site per country. This design permits an assessment of the fidelity of INTE-AFRICA's implementation, a detailed description of the processes, relationships, and contexts involved in the delivery of integrated care, and the identification of factors attributing to

For peer review only - http://bmjopen.bmj.com/site/about/guidelines.xhtml

the failure or success of the programme. It thus addresses the *'black box'* problem in interpreting trial results by improving understanding of the mechanisms that connect particular intervention components to particular outcomes.[50] The chosen approach will enhance social construction and acceptability of chosen decentralised integrated approaches, link outcomes to policy and advocacy and impact sustainability of HIV, DM and hypertension chronic disease service integration in two LMIC countries. It provides the opportunity to document and refine INTE-AFRICA activities prior to a larger pragmatic trial or scale-up by Tanzanian and Ugandan governments. These synergistic approaches to evaluate, understand and respond will support integration, support affordability, address barriers to government scale up and funding barriers for chronic care, inform surveillance, policy and practice development and improve efforts to involve and educate communities in Tanzania and Uganda. It will create a model and a platform of best practices and lessons learnt for other countries implementing integrated and decentralised community health services for HIV and chronic disease. The process evaluation methods for each objective are described in **Table 3**, including how each method maps onto the three different contextual levels.

Table 3: 'Process Evaluation Design and data collection framework'

Study Population and Recruitment

25 patients and 10 health care providers in *Temeke Regional Referral Hospital* in Dar Es Salaam, Tanzania and *Kasangati Health Centre IV*, Kasangati, Uganda will be purposely selected and invited; at six, 12 and 18 months after the start of the trial, to reflect retrospectively on their experience of integration. Health care providers include the hospital/health centre overall in charge and pharmacist, and the '*one stop'* medical officers in-charge, trained clinicians managing HIV, diabetes and hypertension patients, pharmacist, laboratory technician, counsellors or nurses providing health education and counselling, and nurses in the registration desk who are also responsible in taking vital signs from patients. We will also collect qualitative data from interviews with Ministerial policy-makers and provincial/regional/district level clinical/health senior management (directors for NCD, HIV and curative services); NGO and international organisations (for example WHO Country office, UNAIDS, PEPFAR, CDC) and clinical researchers; and conduct FGD with community leaders and community members. These numbers are expected to reach saturation (i.e. the point that further information does not provide any additional variation in observed themes). We may replace participants, for example patients if there is significant loss to follow up or refusal for repeated interviews. For instance, if a participant drops out at 12 months, we still have their six-month experience documented, and we can replace with a new participant, invited to reflect on their 12-month retrospective experience. Where possible we will gender match interviewers with participants (particularly patients). Participants (patients, healthcare providers and others) will not be directly compensated bur rather they will be

For peer review only - http://bmjopen.bmj.com/site/about/guidelines.xhtml

compensated for incurred transport costs to attend the interview/FGD, and provided with refreshments during the interview/FGD.

The following recruitment procedures will take place at each '*one-stop'* integrated care clinic*;*

- Observations will be made in the integrated clinic in consultation with the hospital/health centre and '*one stop'* clinic in-charges.
- Recruitment of patients will be supported by clinic nurses who will identify and approach selected participants who have a minimum of six months experience of integration, and will be asked to consent to partake in an in-depth interview on the day they attend the clinic. INTE-AFRICA researchers will purposively sample women and men of different ages and explore any age/gender and condition ((HIV/hypertensive/DM/multi/co-morbid) related differences.
- *Health care providers* at the integrated clinic will be approached to participate in an in-depth interview on the day the INTE-AFRICA team are scheduled to attend.
- *Ministerial policy-makers and provincial/regional/district level clinical/health senior management* will be identified and requested to participate in a semi–structured interview (face to face, online using Zoom or telephone).
- *NGO and international organisations* will be identified and requested to participate in a semi–structured interview (face to face or telephone).
- *Community leaders* will be identified in the clinic catchment areas by virtue of their position while *community members* will be identified in consultation with the community leaders and invited to participate in the FGDs.
- *Clinical researchers* will be invited to participate in an in depth interview at the 24 month end point.

Data collection

We will use the Empirical Phenomenological Psychological (EPP) five-step method,[51] which combines psychological, interpretative and idiographic components, to collect data. The data will garner an understanding of the complex social processes, of social, aged, gendered and culturally/community specific meanings and broaden the incremental understanding of the distinct lived experience of policy-makers, patients, health care providers, researchers and communities. We will balance the description of phenomena with the interpretation of insights and are cognisant of participant experiential phenomena and authors' interpretation of associated meanings. It will yield an in-depth socio-cultural understanding of patient /participant reported outcomes, their motivations, preferences, beliefs, expectations, identities, hopes and views on conditions, related stigma and of decision-making processes. This will provide better understanding of stakeholder and community positioning during integration. This understanding will inform policy and practice, ensure effective patient/service user

For peer review only - http://bmjopen.bmj.com/site/about/guidelines.xhtml

education, position service users and their families to understand these conditions and interpret study outcomes and facilitate future HIV and chronic disease clinical studies.

Both descriptive patient level data and rich socio-behavioural qualitative/observational data will be collected by a team of trained researchers in Tanzania and Uganda. Data collection will entail exploring the experiences, attitudes and practices of a wide variety of stakeholders during the process of INTE-AFRICA programme implementation and will develop an understanding of the impact of broader structural and contextual factors on the implementation process. [10 45]We will collect data on social behavioural and cultural aspects impacting implementation (e.g. individual and community health risks, protective behaviours and health responses) within the broader social and political frameworks (government resources and barriers to sustaining integration), the practicalities of accessing, providing and sustaining integrated services (e.g. staff time, resources, equity of access, supply chain dynamics and pharmacy components pertaining to drug types, drug/reagent availability and costs for integrated patients, catchment area/populations, quality of care, waiting room dynamics, record keeping and retention across multi/co-morbidities, training gaps); and process indicators (e.g. perceived stigma, acceptability of vertical versus integrated service designs, lay knowledge and awareness, the dynamics of public versus private sector integration (where relevant to the participant), and bottlenecks to accessing services). We will also describe implementation of the intervention in terms of fidelity to the intended model of care, adaptations to the intervention during implementation, and dose and reach of intervention components actually delivered and received (such as numbers and proportions of eligible staff who received integrated care training, numbers of proportions of patient participants who received all or most of their care from integrated services, and frequency of drug stock-outs). The latter data will be complemented by routinely collected quantitative data such as training attendance and medical records. We will document changes in healthcare provider roles, attitudes and patient relationships.

Interviews with patients/service users, healthcare provider and policy-maker/senior manager, will include specific questions about their experience and management of individuals with multi-morbid HIV, hypertension and/or diabetes. These include their perceptions of INTE-AFRICA; impacts of INTE-AFRICA on the provision of integrated HIV/AIDS care and NCD care, and relationships with NGO and international organisations; changes in health provider roles, attitudes, and patient relationships; impacts of the INTE-AFRICA implementation context on trial and health economic cost outcomes; impacts of the INTE-AFRICA intervention on an integrated health systems approach to care (medicine supplies, record keeping, service user education, clinical care pathways, data management, staff training); and barriers to and facilitators of change and future sustainability of integrated care provision. We will assume a more pragmatic approach when garnering perspectives from higher level stakeholders involved in health policy and practice generation, and NGO and international organisations (e.g. WHO country offices, UNAIDS, PEPFAR, CDC) providing peripheral supports and guidance. We

For peer review only - http://bmjopen.bmj.com/site/about/guidelines.xhtml

are especially interested in better understanding the complexities around government scale up and resource allocation for chronic care (for example decentralisation, financial planning, identification of potential funding sources at ministry levels, subsidised NCD drugs, and by international donors (CDC, PEPFAR, UNAIDS). See **Table 3.**

Data analysis and synthesis

The analysis of qualitative data will be iterative, moving between data collection and analysis to test emerging theories. Field notes of observations will be analysed thematically to provide a description of the process and content involved in adapting and delivering the intervention. Audio recordings of interviews and FGDs will be transcribed verbatim by competent and experienced social scientists, with a subsample transcribed using conversation analytic conventions. Translation from local languages (e.g. Swahili, Luganda) into English will be performed for easy sharing with the study partners. Translation will occur using a back-translation method for consistency. An electronic data management package (e.g. NVivo) will be used to manage the qualitative data analysis at the respective country levels. The analysis of the observational data will require knowledge obtained from health professional interviews at different levels to compare how reported experience, and different accounts of patient and professional perspectives relate to actual implementation of INTE-AFRICA scenarios: i) when DM and hypertension services are integrated with HIV-infection services; and ii) comparing countries. Care will be taken to identify and follow up deviant cases which do not fit into emerging theories. Reliability and validity of the analysis is optimised through iterative data collection, the use of a multi-method design incorporating interviews, FGD and observations and the ongoing discussion of findings within the research team for scrutiny and feedback. [52 53]

The chosen phenomenological approach (EPP) to collecting and analysing data, usually used in psychological research, reveals the structures of subjective experience and meaning of a lived phenomenon (in first person point of view). It follows to some extent Husserl's principle of active efforts to *''bracket out''* the researchers' theoretical pre-understanding in the first steps of a text analysis. [51] The ''*bracketing*", however, does not exclude an empathetic, psychological focus in the analysis on the experiences of the researched phenomenon as it is lived by the informant and what it means is to her or him. In the context of INTE-AFRICA, researchers in both countries will strive to get an empathetic understanding of the text, and hence do not apply their professional prior knowledge about integration. The analysis of the observational data will also require knowledge from health professional interviews to compare how reported experience relates to actual implementation of integration at the clinic level.

We will conduct a stepwise EPP analysis in five steps. ***First***, the text will be read several times to get a good grasp of how the informant spoke about the researched phenomenon of integration. In this step,

For peer review only - http://bmjopen.bmj.com/site/about/guidelines.xhtml

theoretical reflection will be withheld. ***Second,*** the whole text will be divided into meaning units of a whole paragraph or a single word. ***Third,*** the informant's personal language will be transformed, unit-by-unit, to the researchers' language. The researchers will discuss the transcription unit by unit. When different interpretations occur, the researchers will return to the interview text and discuss in a free, imaginative process until agreement can be reached through negotiated consensus. ***Fourth***, the text will be screened in a search for comprehensive themes. The text will be interpreted with connection to the researchers' theoretical knowledge in an interchange between the original data, the transformed units and the researchers' theoretical pre-understanding about integration. The meaning units will be assorted into appropriate themes and thus constitute a general structure of the phenomenon of integrated care. ***Fifth,*** this essential structure will penetrate all the revealed themes and thus the meaning of the researched phenomenon of integrated care to the informant.

Credibility and transferability

The process evaluation protocol adheres to recommendations intended to facilitate the standardisation of process evaluation design and reporting [43]. It provides a unique opportunity to document implementation and collaboratively refine integrated care in two sub-Saharan African countries. This makes possible the synthesis of results of similar studies elsewhere in the SSA region in future. In order to ensure credibility, whilst we utilise different methods of data collection (qualitative/observational), and operate concurrently with clinical outcomes data and health economics analysis, we will also add a further layer of triangulation of sources in terms of perspectives across stakeholders and across conditions (HIV/hypertension/DM/multi/comorbidity) when raising the abstraction level. Triangulation of socio-behavioural qualitative and observational data during analysis will occur in order to understand how different types of evidence enhance the overall interpretation of how INTE-AFRICA was implemented, and what the additional health economic and clinical data are, drawing case comparisons across clinics, and across countries, and developing possible explanations for implementation variation. The data, when combined and triangulated across these multi stakeholder perspectives, will provide a '*thick description*', of how the intervention was delivered, maintained and experienced by stakeholders.[10 45] It will also offer explanations for observed variation over time and between countries, and detailed insight into the interaction between different contextual features and components of integration of NCD or HIV/NCD services. It will also facilitate triangulation of information across stakeholders, clinics and countries. This approach will help to support transferability to other settings, by identifying factors which are plausibly and/or consistently related to successful or unsuccessful delivery of intervention components. We recognise the potential for selection and information bias as limitations of the trial itself, and mitigate by using a random sampling approach, defining characteristics in a cohort, using a standardised approach to collecting data with continual assessment of information bias, and ensuring that research personnel are unaware of participant disease status. We will address social desirability in the process evaluation by only providing brief information at the outset of the

For peer review only - http://bmjopen.bmj.com/site/about/guidelines.xhtml

evaluation in order to avoid priming, using an interview schedule approved by a panel of INTE-AFRICA experts in terms of sensitivity, conducting qualitative research using skilled interviewers with limited power relationship between interviewer and participant, conducting the interviews in a safe and secure setting where the participant feels comfortable, briefing them that there is no right and wrong answer, and finally by encouraging them to use anecdotes and experiential evidence to support their views.

Emerging theories and the relationship of the data to the conceptual literature underpinning the intervention will be discussed and refined at INTE-AFRICA research team meetings throughout the project. We envisage utilising the public understanding of science theory [54] to unpack how patients and their communities in Tanzania and Uganda understand and use different knowledge on HIV and NCDs in their lives. This could be facilitated by understanding how they create meaning from scientific findings relating to NCDs and HIV and if, how, and to what degree they incorporate these findings into their everyday lives. This theory has the capacity to shift public attitudes by connecting and communicating the development of innovative scientific concepts in the medical field (in this instance integration of HIV/NCD services in Tanzania and Uganda) to the non-scientific public, and thereby enhance education, training cascade, health policy and practice, and ultimately public understanding of multi-morbidities and sustainable routes to care. Further, it will create a platform for the sharing of lessons learnt, best practices and context adaptation of the final integrated model of care in other African countries (clinical care policies and practice, staff cascade of training, service user education and community awareness raising).

Ethical considerations

Ethical approval for the evaluation has been granted by the research ethics committees of the Liverpool School of Tropical Medicine (UK), the National Institute of Medical Research (Tanzania) and TASO Research Ethics Committee (Uganda). The key ethical principles of voluntary and informed participation, confidentiality and safety of participants will be used in all researcher and participant interactions. Written consent for interviews and observations will be obtained from all participants. All participants will be provided with written information about the research, this will be explained verbally, and informed that their participation is voluntary and that they may withdraw from participation at any time. Safety and confidentiality of all data will be ensured by: (1) encrypting all transcriptions with a password protected code; (2) storing all data in a secure, encrypted database accessible only to authorised persons on the research team; (3) de-linking all personal information of participants from the data collected and stored. Each participant will have a unique identification number.

For peer review only - http://bmjopen.bmj.com/site/about/guidelines.xhtml

Contributorship Statement

All authors contributed to the conceptualisation of the research and contributed to writing the manuscript.

MCVH, MB, JVL, SJ, ES, DB, CP, JO designed the process evaluation protocol.

SJ, MN, SM, JB led the development of the INTE-AFRICA trial.

MCVH drafted the manuscript and all co-authors edited and commented on subsequent drafts. All authors approved the final draft for submission. All authors agree to be accountable for all aspects of the work in ensuring that questions related to the accuracy or integrity of any part of the work are appropriately investigated and resolved.

Funding Statement

The INTE-AFRICA project has received funding from the European Union's Horizon 2020 research and innovation programme under grant agreement No 825698. JVL is supported by a Spanish Ministry of Science, Innovation and Universities Miguel Servet grant (Instituto de Salud Carlos III/ESF, European Union [CP18/00074]) and further acknowledges support to IS Global from the Spanish Ministry of Science, Innovation and Universities through the "Centro de Excelencia Severo Ochoa 2019-2023" Programme (CEX2018-000806-S), and from the Government of Catalonia through the CERCA Programme.

Ethical approval statement

Ethical approval for the evaluation was granted in 2020 at the Liverpool School of Tropical Medicine (UK), the National Institute of Medical Research (Tanzania) and TASO Research Ethics Committee (Uganda).

Data sharing statement

No additional data available.

Competing interests statement

None

Provenance and peer review

Not commissioned; internally peer-reviewed.

For peer review only

Open access

This is an open access article distributed in accordance with the Creative Commons Attribution 4.0 Unported (CC BY 4.0) license, which permits others to copy, redistribute, remix, transform and build upon this work for any purpose, provided the original work is properly cited, a link to the licence is given, and indication of whether changes were made. See: https:// creative commons. org/ licenses/ by/ 4. 0/.

For peer review only

For peer review only - http://bmjopen.bmj.com/site/about/guidelines.xhtml

References

1. Adeloye D, Basquill C. Estimating the prevalence and awareness rates of hypertension in Africa: a systematic analysis. *PLoS One* 2014;9(8):e104300.
2. Atun R, Davies JI, Gale EAM, et al. Diabetes in sub-Saharan Africa: from clinical care to health policy. *Lancet Diabetes Endocrinol* 2017;5(8):622-67.
3. Remais JV, Zeng G, Li G, et al. Convergence of non-communicable and infectious diseases in low- and middle-income countries. *Int J Epidemiol* 2013;42(1):221-7.
4. Ford N, Ball A, Baggaley R, et al. The WHO public health approach to HIV treatment and care: looking back and looking ahead. *Lancet Infect Dis* 2018;18(3):e76-e86.
5. Global, regional, and national comparative risk assessment of 79 behavioural, environmental and occupational, and metabolic risks or clusters of risks, 1990-2015: a systematic analysis for the Global Burden of Disease Study 2015. *Lancet* 2016;388(10053):1659-724.
6. Guwatudde D, Nankya-Mutyoba J, Kalyesubula R, et al. The burden of hypertension in sub-Saharan Africa: a four-country cross sectional study. *BMC Public Health* 2015;15:1211.
7. Lim SS, Vos T, Flaxman AD, et al. A comparative risk assessment of burden of disease and injury attributable to 67 risk factors and risk factor clusters in 21 regions, 1990-2010: a systematic analysis for the Global Burden of Disease Study 2010. *Lancet* 2012;380(9859):2224-60.
8. Addo J, Smeeth L, Leon DA. Hypertension in sub-saharan Africa: a systematic review. *Hypertension* 2007;50(6):1012-8.
9. Chang AY, Gómez-Olivé FX, Manne-Goehler J, et al. Multimorbidity and care for hypertension, diabetes and HIV among older adults in rural South Africa. *Bull World Health Organ* 2019;97(1):10-23.
10. George S, McGrath N, Oni T. The association between a detectable HIV viral load and non-communicable diseases comorbidity in HIV positive adults on antiretroviral therapy in Western Cape, South Africa. *BMC Infect Dis* 2019;19(1):348.
11. Jaffar S, Gill G. The crisis of diabetes in sub-Saharan Africa. *Lancet Diabetes Endocrinol* 2017;5(8):574-75.
12. Jaffar S. Diabetes and other non-communicable diseases in Africa: a potential disaster in the waiting. *Lancet Diabetes Endocrinol* 2016;4(11):875-77.
13. Lupafya PC, Mwagomba BL, Hosig K, et al. Implementation of Policies and Strategies for Control of Noncommunicable Diseases in Malawi: Challenges and Opportunities. *Health Educ Behav* 2016;43(1 Suppl):64s-9s.
14. Katende D, Mutungi G, Baisley K, et al. Readiness of Ugandan health services for the management of outpatients with chronic diseases. *Trop Med Int Health* 2015;20(10):1385-95.
15. Manne-Goehler J, Atun R, Stokes A, et al. Diabetes diagnosis and care in sub-Saharan Africa: pooled analysis of individual data from 12 countries. *Lancet Diabetes Endocrinol* 2016;4(11):903-12.
16. Peck R, Mghamba J, Vanobberghen F, et al. Preparedness of Tanzanian health facilities for outpatient primary care of hypertension and diabetes: a cross-sectional survey. *Lancet Glob Health* 2014;2(5):e285-92.
17. Price AJ, Crampin AC, Amberbir A, et al. Prevalence of obesity, hypertension, and diabetes, and cascade of care in sub-Saharan Africa: a cross-sectional, population-based study in rural and urban Malawi. *Lancet Diabetes Endocrinol* 2018;6(3):208-22.
18. Patel P, Rose CE, Collins PY, et al. Noncommunicable diseases among HIV-infected persons in low-income and middle-income countries: a systematic review and meta-analysis. *Aids* 2018;32 Suppl 1(Suppl 1):S5-s20.
19. Haregu TN, Setswe G, Elliott J, et al. Integration of HIV/AIDS and noncommunicable diseases in developing countries: rationale, policies and models. *International Journal of Healthcare* 2015;1:21-27.
20. Husain NE, Noor SK, Elmadhoun WM, et al. Diabetes, metabolic syndrome and dyslipidemia in people living with HIV in Africa: re-emerging challenges not to be forgotten. *HIV AIDS (Auckl)* 2017;9:193-202.

For peer review only - http://bmjopen.bmj.com/site/about/guidelines.xhtml

21. Juma K, Reid M, Roy M, et al. From HIV prevention to non-communicable disease health promotion efforts in sub-Saharan Africa: A Narrative Review. *Aids* 2018;32 Suppl 1:S63-s73.
22. Haldane V, Legido-Quigley H, Chuah FLH, et al. Integrating cardiovascular diseases, hypertension, and diabetes with HIV services: a systematic review. *AIDS Care* 2018;30(1):103-15.
23. Rabkin M, Melaku Z, Bruce K, et al. Strengthening Health Systems for Chronic Care: Leveraging HIV Programs to Support Diabetes Services in Ethiopia and Swaziland. *J Trop Med* 2012;2012:137460.
24. Some D, Edwards JK, Reid T, et al. Task Shifting the Management of Non-Communicable Diseases to Nurses in Kibera, Kenya: Does It Work? *PLoS One* 2016;11(1):e0145634.
25. Craig P, Dieppe P, Macintyre S, et al. Developing and evaluating complex interventions: the new Medical Research Council guidance. *Bmj* 2008;337:a1655.
26. Iwelunmor J, Ezechi O, Obiezu-Umeh C, et al. Capabilities, opportunities and motivations for integrating evidence-based strategy for hypertension control into HIV clinics in Southwest Nigeria. *PLoS One* 2019;14(6):e0217703.
27. Rachlis B, Naanyu V, Wachira J, et al. Identifying common barriers and facilitators to linkage and retention in chronic disease care in western Kenya. *BMC Public Health* 2016;16:741.
28. Ameh S, Klipstein-Grobusch K, Musenge E, et al. Effectiveness of an Integrated Approach to HIV and Hypertension Care in Rural South Africa: Controlled Interrupted Time-Series Analysis. *J Acquir Immune Defic Syndr* 2017;75(4):472-79.
29. Anand TN, Joseph LM, Geetha AV, et al. Task sharing with non-physician health-care workers for management of blood pressure in low-income and middle-income countries: a systematic review and meta-analysis. *Lancet Glob Health* 2019;7(6):e761-e71.
30. Divala OH, Amberbir A, Ismail Z, et al. The burden of hypertension, diabetes mellitus, and cardiovascular risk factors among adult Malawians in HIV care: consequences for integrated services. *BMC Public Health* 2016;16(1):1243.
31. Edwards JK, Bygrave H, Van den Bergh R, et al. HIV with non-communicable diseases in primary care in Kibera, Nairobi, Kenya: characteristics and outcomes 2010-2013. *Trans R Soc Trop Med Hyg* 2015;109(7):440-6.
32. Golovaty I, Sharma M, Van Heerden A, et al. Cost of Integrating Noncommunicable Disease Screening Into Home-Based HIV Testing and Counseling in South Africa. *J Acquir Immune Defic Syndr* 2018;78(5):522-26.
33. Kwarisiima D, Atukunda M, Owaraganise A, et al. Hypertension control in integrated HIV and chronic disease clinics in Uganda in the SEARCH study. *BMC Public Health* 2019;19(1):511.
34. Fairall LR, Folb N, Timmerman V, et al. Educational Outreach with an Integrated Clinical Tool for Nurse-Led Non-communicable Chronic Disease Management in Primary Care in South Africa: A Pragmatic Cluster Randomised Controlled Trial. *PLoS Med* 2016;13(11):e1002178.
35. Mahomed OH, Asmall S. Development and implementation of an integrated chronic disease model in South Africa: lessons in the management of change through improving the quality of clinical practice. *Int J Integr Care* 2015;15:e038.
36. Matanje Mwagomba BL, Ameh S, Bongomin P, et al. Opportunities and challenges for evidence-informed HIV-noncommunicable disease integrated care policies and programs: lessons from Malawi, South Africa, Swaziland and Kenya. *Aids* 2018;32 Suppl 1:S21-s32.
37. Nuche-Berenguer B, Kupfer LE. Readiness of Sub-Saharan Africa Healthcare Systems for the New Pandemic, Diabetes: A Systematic Review. *J Diabetes Res* 2018;2018:9262395.
38. Pfaff C, Scott V, Hoffman R, et al. You can treat my HIV - But can you treat my blood pressure? Availability of integrated HIV and non-communicable disease care in northern Malawi. *Afr J Prim Health Care Fam Med* 2017;9(1):e1-e8.
39. Pfaff C, Singano V, Akello H, et al. Early experiences integrating hypertension and diabetes screening and treatment in a human immunodeficiency virus clinic in Malawi. *Int Health* 2018;10(6):495-501.

For peer review only - http://bmjopen.bmj.com/site/about/guidelines.xhtml

40. Rawat A, Uebel K, Moore D, et al. Integrated HIV-Care Into Primary Health Care Clinics and the Influence on Diabetes and Hypertension Care: An Interrupted Time Series Analysis in Free State, South Africa Over 4 Years. *J Acquir Immune Defic Syndr* 2018;77(5):476-83.
41. Jaffar S, Amuron B, Foster S, et al. Rates of virological failure in patients treated in a home-based versus a facility-based HIV-care model in Jinja, southeast Uganda: a cluster-randomised equivalence trial. *Lancet* 2009;374(9707):2080-89.
42. Georgeu D, Colvin CJ, Lewin S, et al. Implementing nurse-initiated and managed antiretroviral treatment (NIMART) in South Africa: a qualitative process evaluation of the STRETCH trial. *Implement Sci* 2012;7:66.
43. Moore G, Audrey S, Barker M, et al. Process evaluation in complex public health intervention studies: the need for guidance. *J Epidemiol Community Health* 2014;68(2):101-2.
44. Linnan L, Steckler A. Process evaluation for public health interventions and research. An overview. In: Steckler A, Linnan L, eds. Process evaluation for public health interventions and research. 1st ed. San Francisco: Josset-Bass 2002:1-23.
45. Murdoch J, Curran R, Bachmann M, et al. Strengthening the quality of paediatric primary care: protocol for the process evaluation of a health systems intervention in South Africa. *BMJ Glob Health* 2018;3(Suppl 5):e000945.
46. Kippax S. Effective HIV prevention: the indispensable role of social science. *J Int AIDS Soc* 2012;15(2):17357.
47. Bronfenbrenner U. Toward an experimental ecology of human development. *American Psychologist* 1977;32(7):513-31.
48. Bronfenbrenner U. The ecology of human development: Experiments by nature and design. Cambridge, MA: Harvard University Press 1979.
49. Hawe P, Shiell A, Riley T. Theorising interventions as events in systems. *Am J Community Psychol* 2009;43(3-4):267-76.
50. Grimshaw JM, Zwarenstein M, Tetroe JM, et al. Looking inside the black box: a theory-based process evaluation alongside a randomised controlled trial of printed educational materials (the Ontario printed educational message, OPEM) to improve referral and prescribing practices in primary care in Ontario, Canada. *Implement Sci* 2007;2:38.
51. Karlsson G. Psychological Qualitative Research From a Phenomenological Perspective: Almqvist & Wiksell International 1993.
52. Marshall C, Rossman GB. Designing qualitative research. 4th ed. Thousands Oaks, Calif: Sage Publications 2006.
53. Patton MQ, Patton MQ. Qualitative research and evaluation methods. Thousand Oaks, Calif.: Sage Publications 2002.
54. Brossard D, Lewenstein B. A Critical Appraisal of Models of Public Understanding of Science: Using Practice to Inform Theory2010:11-39.

For peer review only

For peer review only - http://bmjopen.bmj.com/site/about/guidelines.xhtml

Table 1 'Key data on the country settings'

	Tanzania	Uganda
Income level	Low	Low
Population size	58m (2018)	35m (2016)
Estimated prevalence of hypertension from STEPS survey	26%	26%
Estimated prevalence of diabetes from STEPS survey [15] *	5-10%	2-5%
Estimated prevalence of HIV-infection	5.1% (2017)	6.2% (2017)
Doctors density /100,000 population	3 (2014)	0.8 (2005)

* diabetes estimate varies according to age and gender. Data are of variable quality but reference 11 shows that the overall median diabetes prevalence in 12 countries in Africa is 5%.

For peer review only

For peer review only - http://bmjopen.bmj.com/site/about/guidelines.xhtml

Table 2 'Logic Model of programme inputs, processes and outcomes'

Intervention inputs	Changes in care processes	Outcomes
Negotiation with national, district and local government health departments, NGOs and funders	Agreement about and support for service model, including reorganisation of clinics and staff<br>Commitment to ensure drug supply	• Reorganisation of clinics and staff to implement the model<br>• An effective, quality, and sustainable funded drug supply chain
Negotiating lower drug prices<br>Supporting and monitoring drug ordering in each clinic<br>Providing buffer drug supplies to each clinic	Drugs always in stock	• Increased diagnosis of comorbid conditions<br>• Increased retention and adherence<br>• Increased viral suppression, better control of blood pressure and blood glucose<br>• Less AIDS, cardiovascular disease and diabetes complications<br>• Lower patients costs (travel and absence from work)<br>• Less health service duplication and costs (health service costs might increase if more patients are diagnosed and are more adherent)<br>• Increase patient satisfaction<br>• Increased clinician satisfaction; less burn-out and absenteeism<br>• Reduce missed opportunities for improving care and health outcomes
Engagement with and support for clinicians and managers in each clinic	Clinicians and managers enable and support integration, and find solutions to emerging problems	
Provision of integrated service in each clinic (alongside and additional to existing services)	Trial participants attend integrated service<br>Avoid multiple visits for patients with multi-morbidity	
Training clinicians about integrated clinical management	Better diagnosis and treatment including attention to comorbid conditions	
Community engagement	Identify and enlist community organisations and resources to help with health education, tracing defaulters or patients who have difficulty attending clinic	
Providing standardised stationery for integrated medical records; training clinicians to use it	Increased awareness by clinicians and patients about disease severity, comorbidity, adherence and control in individual patients	
Improving monitoring and evaluation based on clinics registers and medical records	Regular data analysis and feedback to staff	Quality assurance and continuous improvement in the quality of care
Identifying effective health education	Improve health education at clinics	Healthier lifestyles<br>Increased adherence

For peer review only

For peer review only - http://bmjopen.bmj.com/site/about/guidelines.xhtml

Table 3: 'Process Evaluation Design and data collection framework'

**Post Integration**<br>*** Numbers indicated are per country/.**<br>**Data Collection at each Site**	**6 months**	**12 months**	**18 months**	**Contextual level**
Observations of consultations, different processes and clinic flow at clinic levels and in non-clinical areas.	1 week	1 week	1 week	**Micro context (facility and neighbourhoods)**
In depth phenomenological interviews with patients/service users	25	25	25	**Micro context (facility and neighbourhoods)**
In depth phenomenological interviews with health care providers at the clinic (hospital overall in charge, hospital pharmacist, the medical officers in-charge of the integrated clinic, trained clinicians managing HIV, diabetes and hypertension patients, pharmacist, laboratory technician, counsellors or nurses providing health education and counselling and nurses in the registration desk who are also responsible in taking vital signs)	10	10	10	**Meso context (national to regional/city)**<br>**Micro context (facility and neighbourhoods)**
Semi-structured interviews with Ministerial policy-makers and provincial/regional/district level clinical/health senior management (Director for NCD, HIV and curative services).	-	5	5	**Macro context (Global)**<br>**Meso context (national to regional/city)**
Semi-structured interviews with NGO and international organisations (for example WHO Country office, UNAIDS, PEPFAR, CDC)	-	5	5	**Macro context (Global)**<br>**Meso context (national to regional/city)**
Focus group discussions (FGD) with community leaders (8-12 participants)	1	1	1	**Micro context (facility and neighbourhoods)**
Focus group discussions (FGD) gender specific with community members(8-12participants)	2	2	2	**Micro context (facility and neighbourhoods)**
In depth phenomenological interviews with clinical researchers			4	**Micro context (facility and neighbourhoods)**<br>**Meso context (national to regional/city)**

For peer review only

Figure One ‘Potential benefits of integrating diabetes, hypertension and HIV services for a) DM and hypertension control, and b) HIV control’

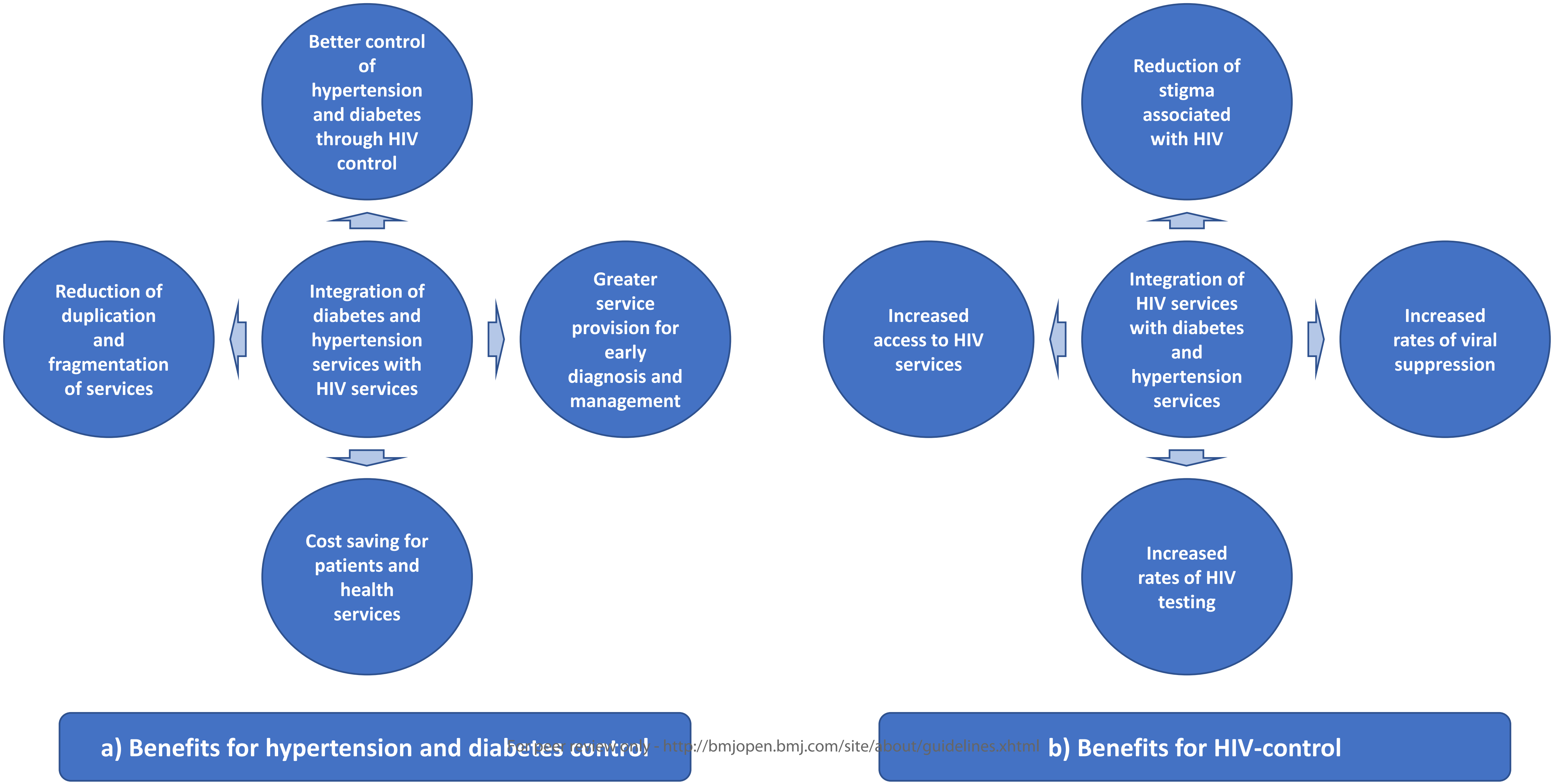

For peer review only - http://bmjopen.bmj.com/site/about/guidelines.xhtml

Figure Two 'INTEAFRICA Conceptual Model'

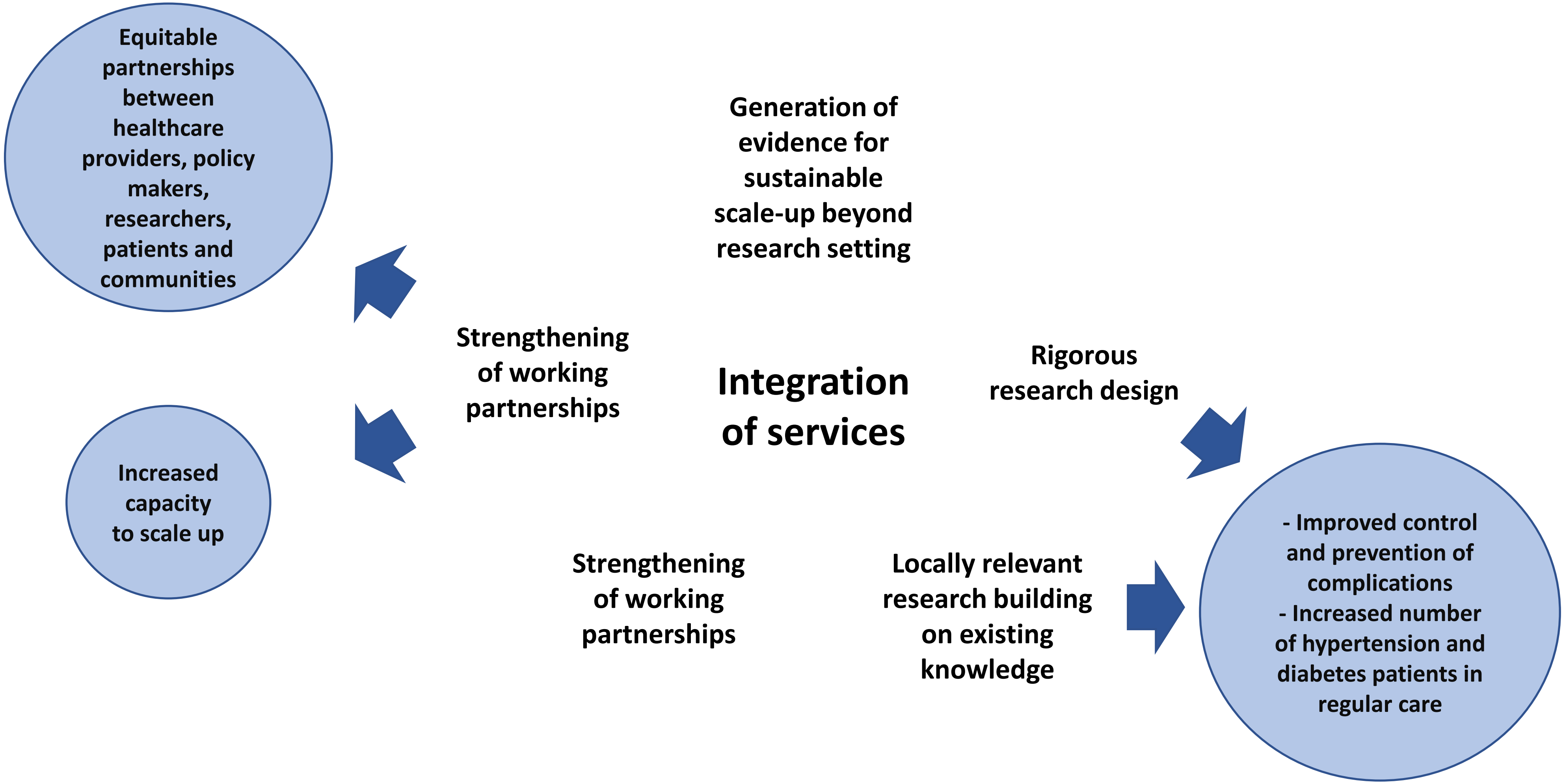

Figure Three ‘Potential contextual influences on INTEAFRICA programme implementation cascade’

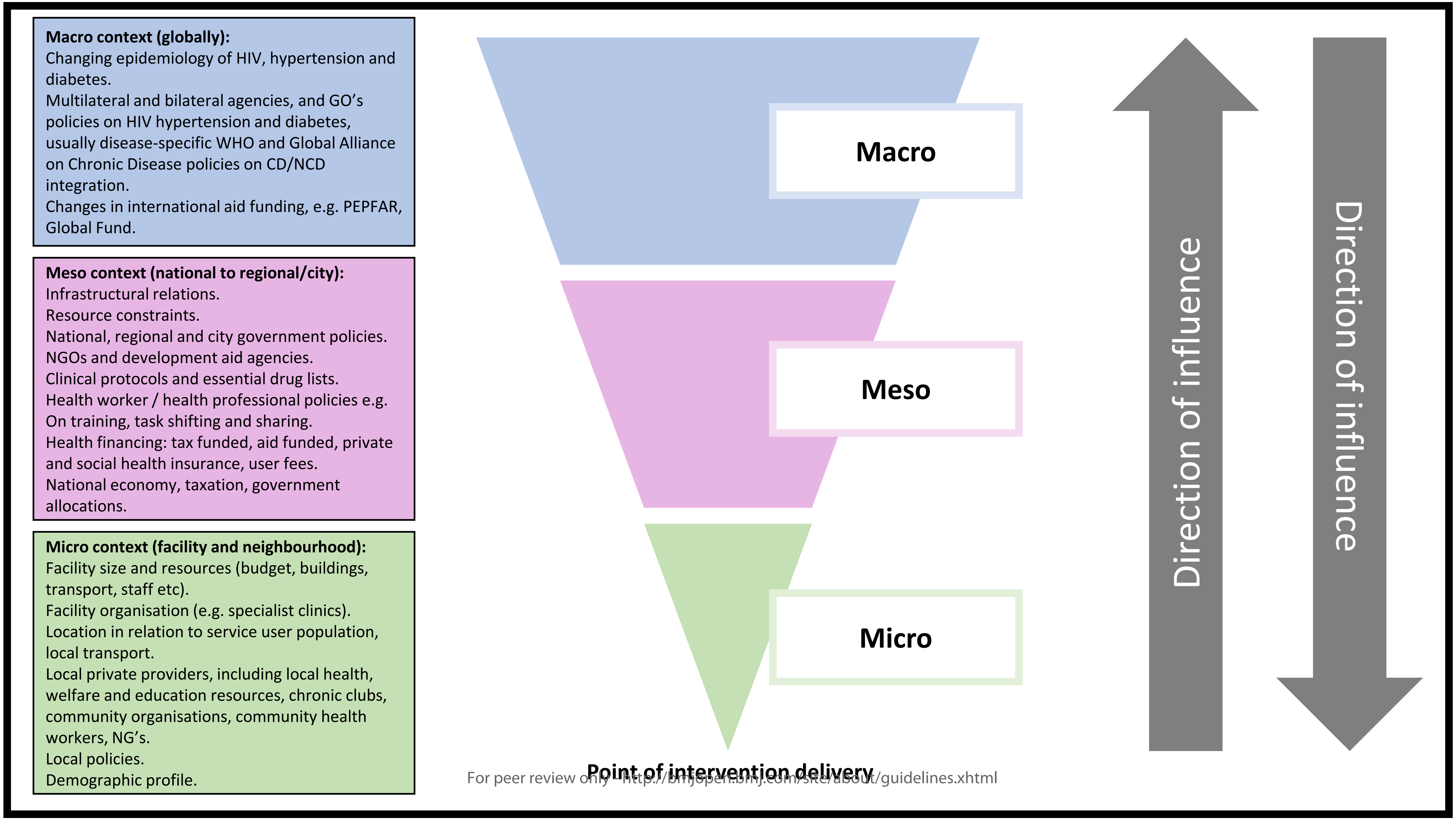

For peer review only - http://bmjopen.bmj.com/site/about/guidelines.xhtml
